# Concentration-dependent nitrogen functionalization of graphene nanoplatelets: structural, surface, and electrical properties

**DOI:** 10.1039/d6ra08002b

**Published:** 2026-11-03

**Authors:** Rashida Huseynzade, Mustafa Muradov, Sevinj Hamidova, Ali Guseinov, Khatira Mehtiyeva, Mariya V. Dobrotvorska, Mikołaj Lewandowski, Alfonso Policicchio, Lorenzo Caputi, Ákos Kukovecz, Stefano Bellucci, Lala Gahramanli

**Affiliations:** a Faculty of Physics, Chemical Physics of Nanomaterials, Baku State University Zahid Khalilov 33 Baku AZ1148 Azerbaijan lalagahramanli@bsu.edu.az; b Nanoresearch Laboratory, Center of Excellence, Baku State University Zahid Khalilov 33 Baku AZ1148 Azerbaijan; c Faculty of Physics, Physics of Semiconductor, Baku State University Zahid Khalilov 33 Baku AZ1148 Azerbaijan; d Advanced Material Science R&D Center, Baku State University Zahid Khalilov 33 Baku AZ1148 Azerbaijan; e NanoBioMedical Centre, Adam Mickiewicz University Wszechnicy Piastowskiej 3 Poznań 61-614 Poland; f Department of Physics, University of Calabria Via P. Bucci, Cubo 33C I-87036 Rende Italy; g Department of Applied and Environmental Chemistry, University of Szeged Rerrich B. 1 H-6720 Szeged Hungary; h Qi S.r.l. Via Monte d'Oro, 2/A 00071 Pomezia (RM) Italy

## Abstract

Nitrogen-functionalized graphene nanoplatelets (N-GNPs) were synthesized by a low-temperature dopamine/HCl-assisted microwave method using 1–5% nominal N precursor concentrations to investigate concentration-dependent structural evolution. The materials were characterized by SEM, TEM, EDX, XRD, FTIR, Raman, XPS, BET analysis, and electrical measurements. SEM revealed thin, wrinkled multilayer graphene sheets suitable for N functionalization. XRD confirmed preservation of the graphitic framework, while the interlayer spacing changed non-monotonically from 0.33724 nm (pure) to 0.33924 nm (1%) and 0.33576 nm (5%), reflecting competition between N-induced expansion and π–π restacking. FTIR confirmed the formation of C–N/C=N and N–H functional groups. Raman analysis showed maximum defect generation for the 2% sample, where the *I*_D_/*I*_G_ ratio increased from 0.243 to 0.492, and the apparent graphitic domain size decreased from 79.1 to 39.1 nm, while *I*_2D_/*I*_G_ < 1 confirmed preservation of the multilayer structure. XPS N 1s spectra revealed two components at binding energy ∼399.6 and ∼401.5 eV, attributed to pyrrolic/amine-type and graphitic N or/and/or protonated amine species, respectively, supporting N modification of the GNP surface. BET analysis showed the highest specific surface area (45.6 m^2^ g^−1^) for the GNP-N 2% sample. Electrical measurements revealed a non-linear dependence on N content, with N-GNPs 2% showing the highest conductivity among the functionalized samples (72.73 S cm^−1^), although remaining below pure GNPs (88.50 S cm^−1^). Combined with its maximum BET surface area, this composition (2%) provides the most favorable balance among the measured surface, structural, and electrical properties within the N-functionalized series.

## Abstract 2

N-GNPs were synthesized by a low-temperature dopamine/HCl-assisted microwave method using 1–5% nominal N precursor concentrations to investigate concentration-dependent structural and physical evolution.

## Graphical abstract

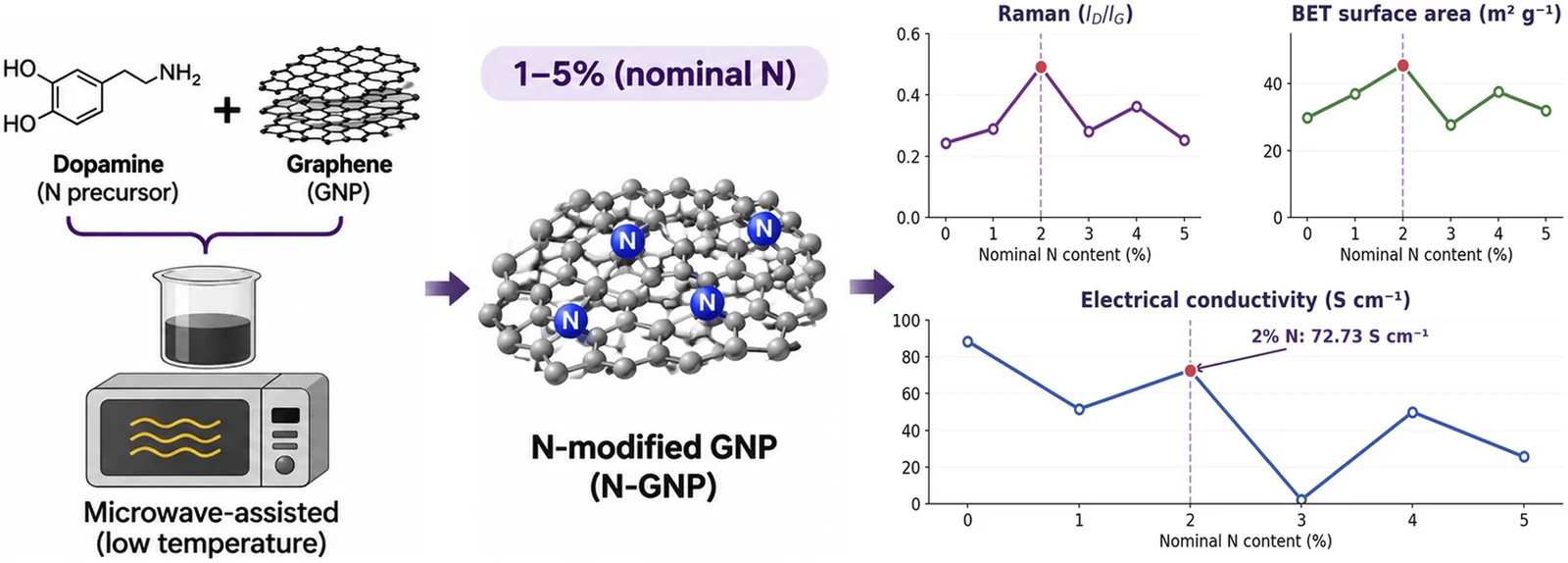

## 1. Introduction

Graphene is a two-dimensional sp^2^-hybridized carbon (C) material consisting of a hexagonal network of C atoms. Its high carrier mobility, large specific surface area, high thermal conductivity, and mechanical strength make it attractive for electronic, sensing, catalytic, energy storage, water treatment, and composite applications.^1,2^ In real materials, however, graphene is often present as few-layer or multilayer nanoplatelets rather than as isolated monolayers. The number of layers, stacking order, wrinkles, edges, lateral size distribution, and interflake contacts strongly influence the measured properties. This is especially important for graphene nanoplatelets (GNPs), where macroscopic electrical, thermal, and adsorption performance does not simply reflect an ideal monolayer.

Using expandable graphite and liquid-phase exfoliation is attractive because it offers a scalable route to graphene nanoplatelets. This route also makes the chemical interpretation more complex than for ideal monolayer graphene: the final material is an assembly of stacked flakes with edges, wrinkles, residual defects, and interflake contacts. Literature on liquid exfoliation, functionalized GNPs, and pressed GNP networks shows that processing history controls both the density of accessible reactive sites and the macroscopic response of the resulting material.^3–7^ For this reason, the present data should be read as the response of a real multilayer GNP system, not as the response of an isolated perfect graphene sheet.

One limitation of pure graphene is its zero-gap semi-metallic electronic structure. Chemical modification and heteroatom incorporation are widely used to tune charge density, surface chemistry, and chemical activity. Recent studies have demonstrated that tailoring the structure, interlayer interactions, and surface chemistry of graphene, metal oxides, and transition metal chalcogenides provides an effective strategy for engineering their physicochemical properties and broadening their potential applications.^8,9^ N is particularly important because it has one more valence electron than C and can generate several bonding environments in graphitic C, commonly described as pyridinic, pyrrolic, graphitic/substitutional, amine-like, and oxidized N.^4,10–14^ These configurations do not have equivalent effects: edge-type N mainly changes defect chemistry, polarity, and surface reactivity, while graphitic N can more directly modify the basal-plane electronic structure. This distinction is central for avoiding overinterpretation of FTIR and Raman data.

For a dopamine/HCl treatment carried out at moderate temperature, it is scientifically safer to describe the product as N-modified or N-functionalized GNP unless direct chemical-state evidence is available. Dopamine and polydopamine are widely used as adhesives and N-containing surface modifiers because dopamine can polymerize and deposit on a wide range of substrates, creating amine, imine, catechol/quinone, and related interfacial motifs.^15,16^ On graphitic materials, these species are expected to interact preferentially with edges, defects, oxygenated residues, and wrinkled regions. Thermal or microwave treatment can then convert part of this interfacial chemistry into C–N/C=N-containing structures, but Raman, FTIR, and XRD alone cannot quantify the fraction of substitutional graphitic N.

Raman spectroscopy, XRD, and FTIR provide complementary but different levels of structural information. Raman spectroscopy probes disorder, strain, doping effects, layer number, and the evolution of the D, G, and 2D bands.^17–23^ XRD, on the other hand, evaluates graphitic stacking and interlayer spacing, while FTIR reveals changes in local bonding and surface functional groups. High-resolution XPS, especially analysis of the N 1s region, is normally required to distinguish pyridinic, pyrrolic, graphitic, and oxidized N with confidence.^4,12–14^ The strength of the present dataset is therefore not the chemical-state quantification, but the internally consistent combination of preserved graphitic stacking with altered local bonding and Raman defect activation.

Unlike previous studies on dopamine-derived N modification of GNP, this work provides a comprehensive and correlated structural analysis by combining XRD, FTIR, Raman, XPS, and BET characterization to elucidate how the nominal dopamine concentration controls the evolution of defects, N incorporation, and accessible surface area in multilayer GNPs. Rather than relying on a single characterization technique, the study establishes clear structure–property relationships through complementary experimental evidence, enabling a more reliable interpretation of N-induced structural changes. Furthermore, a deliberately conservative interpretation is adopted by distinguishing N functionalization from unequivocal substitutional graphitic N, thereby providing a rigorous framework for assessing dopamine-derived N modification in multilayer GNPs.

### 1.1. Broader literature framework

Several groups have shown that N incorporation and N-rich functionalization can tune graphene properties, but the reported response depends strongly on the synthesis route. High-temperature ammonia treatment, plasma treatment, CVD, and arc-discharge methods can produce more deeply incorporated N species, whereas wet-chemical and polymer-derived routes often emphasize edges, defects, and surface functionalities.^4,10,11,14,24–26^ The present dopamine/HCl route is more closely associated with the latter category; therefore, N modification is a more appropriate description than uniform substitutional doping.

The GNP-specific literature adds another important constraint. Functionalized graphene nanoplatelets can carry hydroxyl, amino, carboxyl, imine, or other terminal groups while retaining the stacked graphitic scaffold.^5–7^ In such materials, the property change is rarely controlled by one variable only. Edge chemistry, platelet size, stacking, wrinkling, residual oxygen (O), trapped solvent, contact resistance, and flake orientation can all contribute. This explains why the present XRD, FTIR, and Raman trends need not be monotonic with nominal dopamine concentration.

Earlier GNP studies, including work on liquid exfoliation, thermal diffusivity, microwave contacts, GNP/Cu nanoparticle hybrids, conductive polymer composites, and pollutant filters, remain useful here, but should be used as contextual evidence rather than as the main narrative. They show that GNP systems behave as networks of interacting platelets and that practical performance depends on interflake connectivity, packing, and accessible surface chemistry.^3,27–32^ This is precisely the materials framework needed for interpreting N-modified GNPs.

Dopamine-derived chemistry provides a second literature axis. Polydopamine coatings are known for substrate-independent adhesion and for creating N-containing interfacial layers.^15,16^ When dopamine is used with graphene-based materials, the resulting product may contain deposited or grafted N-rich species, partial reduction/oxidation effects, and surface-bound polymeric residues. Therefore, the observed FTIR changes should be interpreted as evidence of altered local chemistry, but not as a conclusive evidence proof of a single N configuration.

The third axis is Raman defect physics. The *I*_D_/*I*_G_ ratio is a powerful indicator, but it is not a simple linear counter of all defects over all disorder regimes.^20–23^ Edge defects, vacancy-like defects, sp^3^-type defects, substitutional heteroatoms, and domain boundaries can activate the D band differently. In multilayer nanoplatelets, the measured Raman response can also be affected by orientation, restacking, local heating, background subtraction, and sampling position. The non-monotonic *I*_D_/*I*_G_ trend in the present series is therefore plausible and should be discussed as a competition between defect creation, functional-group rearrangement, and network-level changes.

The novelty of the presented study is in establishing a concentration-dependent relationship between N functionalization at different concentration levels (1–5%) and the structural, surface, and electrical properties of GNPs. Particular attention is given to identifying a functionalization level that provides a favorable balance between defect generation, accessible surface area, and electrical transport while preserving the multilayer graphitic framework.

## 2. Experimental

### 2.1. Materials

Expandable graphite (99%, Grade 1721, Asbury Carbon/Asbury Carbons, USA) was used as the starting C source. Isopropyl alcohol (IPA, C_3_H_8_O, ≥99.0%, Sigma-Aldrich) was used as the dispersion solvent. Dopamine hydrochloride (≥98%, Merck or Sigma-Aldrich) was used as the N-containing precursor. Hydrochloric acid (HCl, 37 wt%, Merck, Germany) and ethanol (C_2_H_6_O, ≥99.8%, Sigma-Aldrich, USA). HCl was added to adjust the pH and promote dopamine interaction with the graphene surface, and C_2_H_6_O was used during the washing step to remove excess precursor and unreacted residues.

### 2.2. Preparation of GNPs

GNPs were prepared from expandable graphite by liquid-phase ultrasonication. In a typical preparation, 0.2 g of expandable graphite was dispersed in 200 mL of IPA and treated with ultrasonic waves for 1–2 h. The suspension was filtered, and the obtained solid was washed 4–5 times with IPA. The final product was dried in a vacuum oven at 100 °C for 24 h. A schematic representation of the preparation of pure GNPs are shown in Fig. 1.

**Fig. 1:**
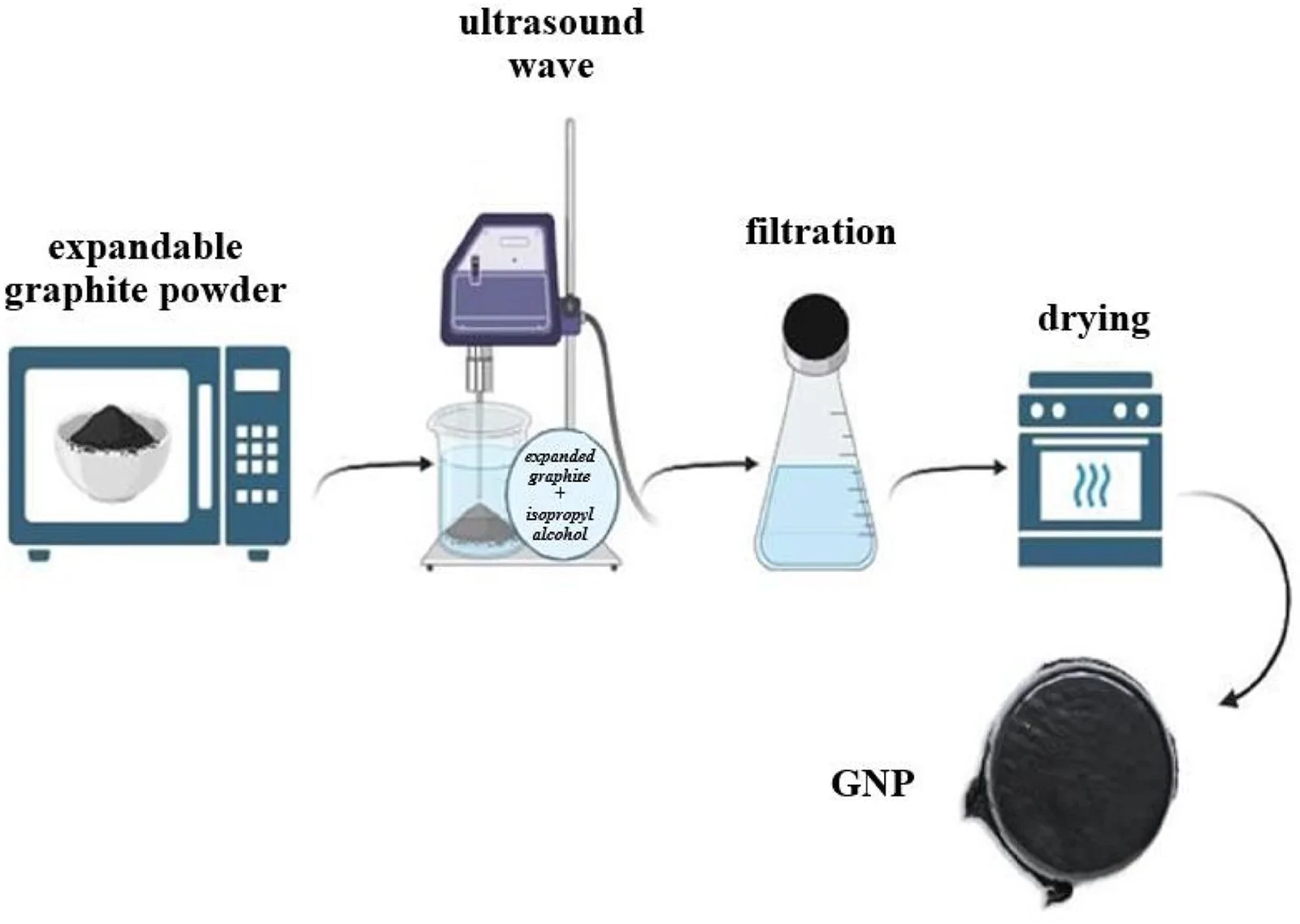
Schematic representation of the preparation of pure GNPs.

### 2.3. Preparation of N-functionalized GNPs

Dopamine was used as the N source. First, 0.5 g of GNPs was added to 100 mL of IPA and sonicated in an ultrasonic bath for 1 h. Dopamine/HCl precursor solutions were prepared at nominal N-modification levels of 1, 2, 3, 4, and 5%. HCl was added gradually at 5–10 min intervals until the pH reached 4–5, and the mixture was stirred in the dark. The dopamine/HCl solution was then added slowly to the GNP dispersion over 15 min and sonicated for 1 h at 25 °C. After dispersion, the sample was treated in a microwave oven (750 W) for 150 s, cooled to room temperature, washed 3–4 times with ethanol using filter paper, and dried at 150 °C for 3 h. The schematic representation of the GNPs N-modification route using dopamine/HCl treatment is shown in Fig. 2.

**Fig. 2:**
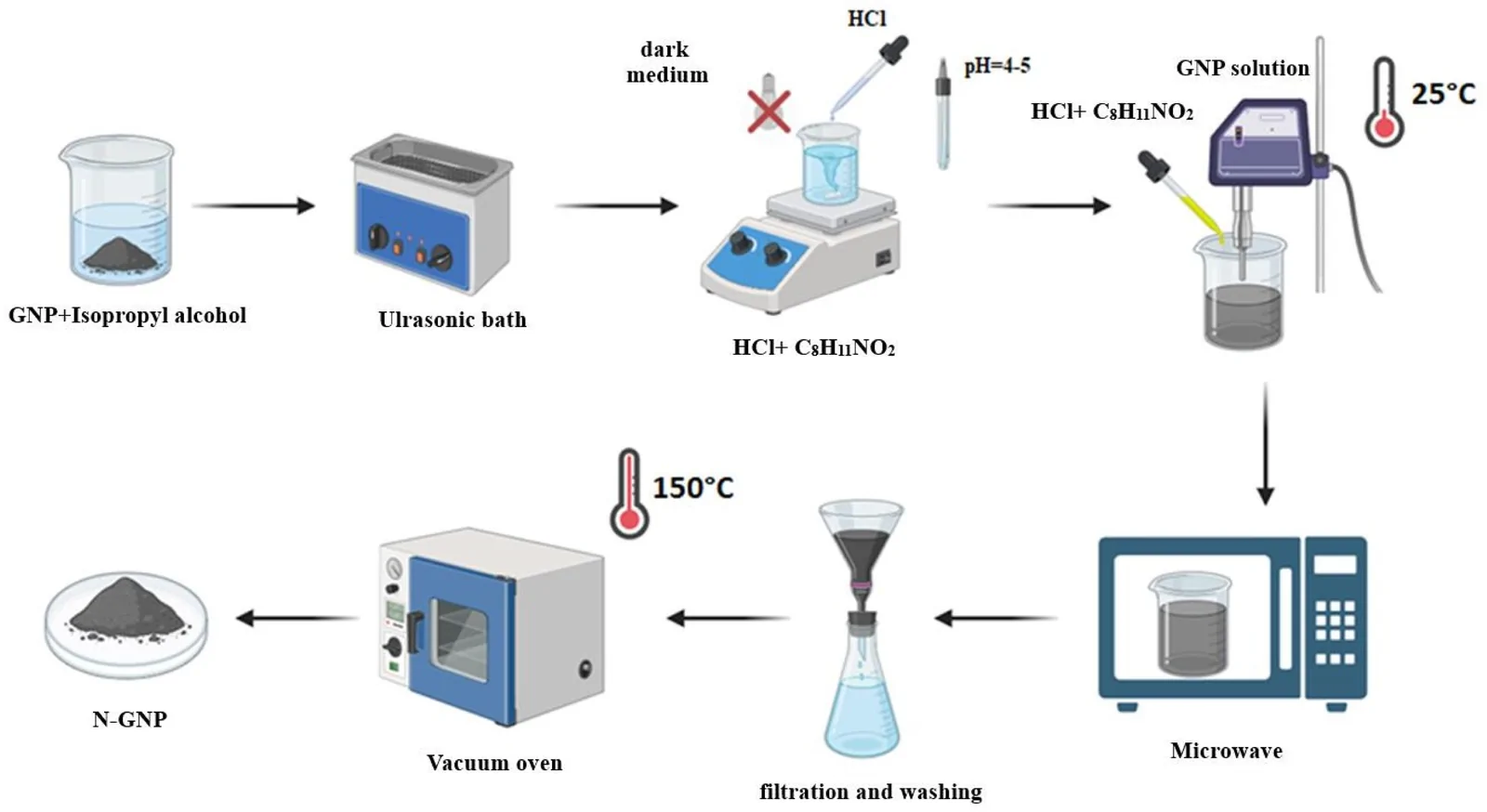
Schematic representation of the GNPs N-functionalized route using dopamine/HCl treatment.

Dopamine was used as the N-containing precursor for the preparation of N-functionalized GNPs. The samples were prepared at nominal N-modification levels of 1, 2, 3, 4, and 5%. The corresponding volumes of dopamine solution and HCl used for each nominal modification level are presented in Table 1. The reagent volumes were proportionally increased relative to those used for the 1% sample according to:

1 *V*_*n*_ = *nV*_1%_

where *V*_*n*_is the reagent volume required for the selected nominal modification level, *V*_1%_is the corresponding volume used for the 1% sample, and *n* is the nominal modification level (1–5%).

**Table 1:** Volumes of dopamine solution and HCl used for the preparation of GNPs at different nominal N-modification levels

Samples	Dopamine, mL	HCl, mL
N-GNPs 1%	2.5	0.69
N-GNPs 2%	5	1.37
N-GNPs 3%	7.5	2.06
N-GNPs 4%	10	2.74
N-GNPs 5%	12.5	3.43

The resulting samples are denoted N-GNPs 1%, N-GNPs 2%, N-GNPs 3%, N-GNPs 4%, and N-GNPs 5%.

The synthesized N-functionalized GNP samples were washed approximately 6–7 times with ethanol to remove residual dopamine, HCl, unreacted species, and reaction by-products. The washing process was carried out using filter paper and repeated until a pH of approximately 5.5–6.0 was reached. In the final washing step, IPA was added to the samples. After sedimentation, the samples were carefully transferred into Petri dishes and subsequently dried in an oven at 150 °C for 3 h.

The primary mechanism for N-functionalization of GNPs using dopamine can be described as follows: dopamine first transforms into a polydopamine (PDA) layer on the graphene surface; subsequently, upon heating, this layer integrates into the C network, resulting in N-functionalization.^33–35^ Central to this mechanism is a sequence involving dopamine oxidation, quinone formation, cyclization, the generation of 5,6-dihydroxyindole (DHI), and subsequent polymerization. As a result, N is incorporated in pyrrolic, pyridinic, and sometimes graphitic forms.^34,35^ The mechanism of N-functionalization of GNPs is shown in Fig. 3.

**Fig. 3:**
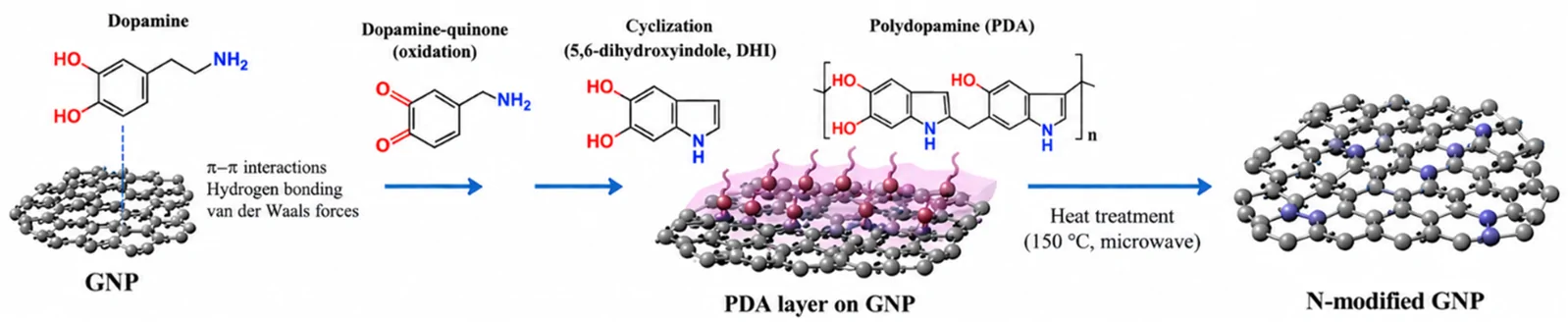
The mechanism of N functionalization GNP.

As shown in Fig. 3, dopamine adsorbs onto the graphene surface; this is primarily attributed to π–π interactions, hydrogen bonding, and van der Waals forces. Dopamine is subsequently oxidized to dopamine–quinone, which yields DHI through intramolecular cyclization and rearrangement, followed by polymerization to form PDA. The N-containing functional groups inherent in PDA are subsequently incorporated into the graphene-derived C framework during carbonization.^36–39^ Upon heating, the PDA layer undergoes partial carbonization, resulting in the formation of N-modified graphene.^36–39^

To evaluate the reproducibility of the synthesis and characterization results, all samples were independently prepared in two separate batches. The structural characterizations were repeated for both batches, yielding consistent results within the experimental uncertainty.

### 2.4. Characterization methods

Phase and structural analysis were carried out using a Bruker D8 ENDEAVOR powder X-ray diffractometer equipped with a LYNXEYE detector. Measurements were performed in Bragg–Brentano geometry using Cu Kα radiation (*λ* = 1.5406 A). Fourier-transform Infrared (FTIR) spectra were acquired using an IR Affinity spectrometer (Shimadzu) in the range 400–4000 cm^−1^. Raman spectra were collected using an EnSpectr R532 Raman spectrometer with 532 nm laser excitation. Morphology was examined by Scanning Electron Microscopy (SEM) using an FEI Quanta Inspect, tungsten filament, 25 kV, and EDX analysis. The morphology of the samples was examined with a JEM-1400 Transmission Electron Microscope (TEM) (JEOL, Japan) operated at an accelerating voltage of 80–120 kV. X-ray photoelectron spectroscopy (XPS) measurements were performed in an Omicron ultra-high-vacuum system using an Mg Kα source (1253.6 eV) and a hemispherical electron energy analyzer. The solutions of the samples in propanol were drop-cast onto silicon substrates covered with a thin layer of gold. The region spectra were obtained with a pass energy (PE) of 20 eV, and the survey scans with PE = 50 eV. The fitting procedure was performed by applying a Shirley background subtraction and using line shapes modeled as a linear combination of Gaussian and Lorentzian functions (GL30) or asymmetric Lorentzian (LA(1.2,2.5,5)). All the spectra were calibrated to the position of the Au 4f_7/2_ line at 84.0 eV. The measurement of the specific surface area of the samples was determined by low-temperature N adsorption by the multipoint BET method on a SORBI-MS instrument (ZAO META). The Brunauer–Emmett–Teller (BET) specific surface areas were measured from N adsorption data. Electrical contacts were formed using silver paste in a planar configuration. The current–voltage (*I*–*V*) characteristics were recorded to evaluate the electrical transport properties of the samples.

## 3. Results and discussion

### 3.1. Morphological and elemental analysis

The SEM images of pure GNPs show thin, wrinkled, and overlapped platelets with a clear sheet-like morphology in Fig. 4. Wrinkles and folded edges are typical of exfoliated GNPs and indicate that the material is not a compact graphite powder, but a multilayer platelet assembly. The wrinkled surface is relevant because edges, folds, and pre-existing defects are the most likely anchoring sites for dopamine-derived N-containing groups.

**Fig. 4:**
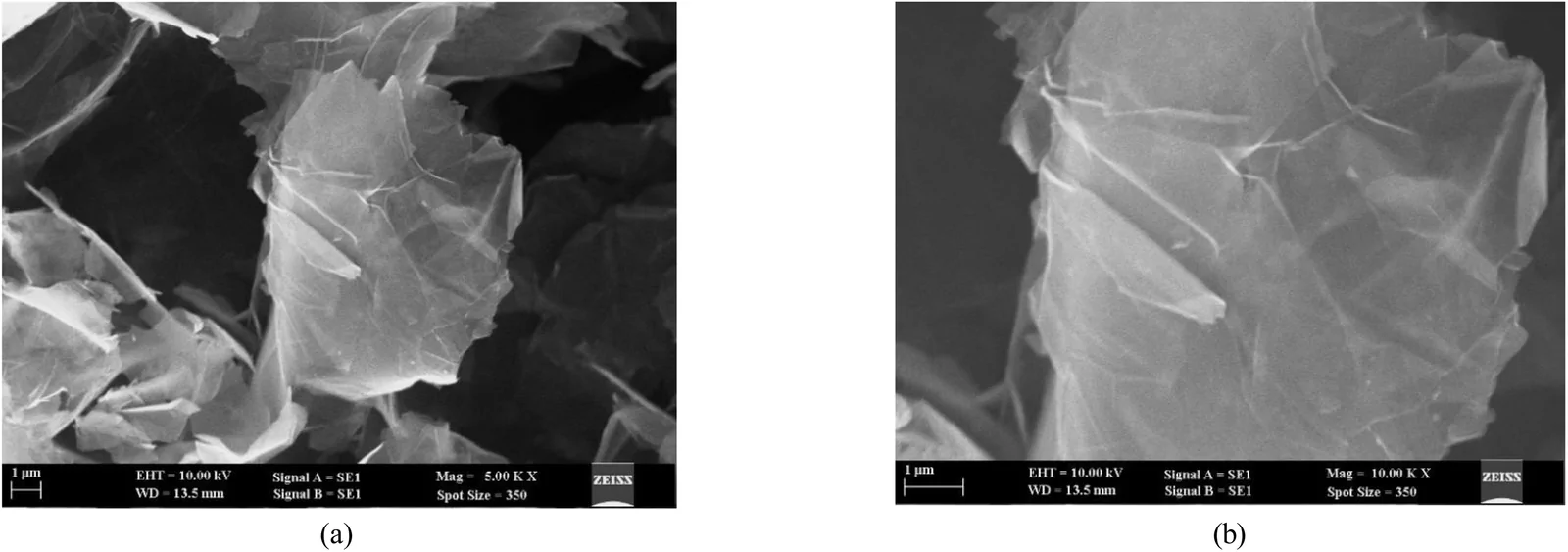
SEM images of pure GNPs at different magnifications (a-5.00 kx and b-10.00 kx).

The SEM morphology is also consistent with application-oriented GNPs literature. Pressed GNP filters and GNP contacts behave as networks of partially restacked platelets rather than continuous monolayer sheets.^27–29^

TEM and EDX analyses of pure GNPs and N-functionalized GNPs are shown in Fig. 5.

**Fig. 5:**
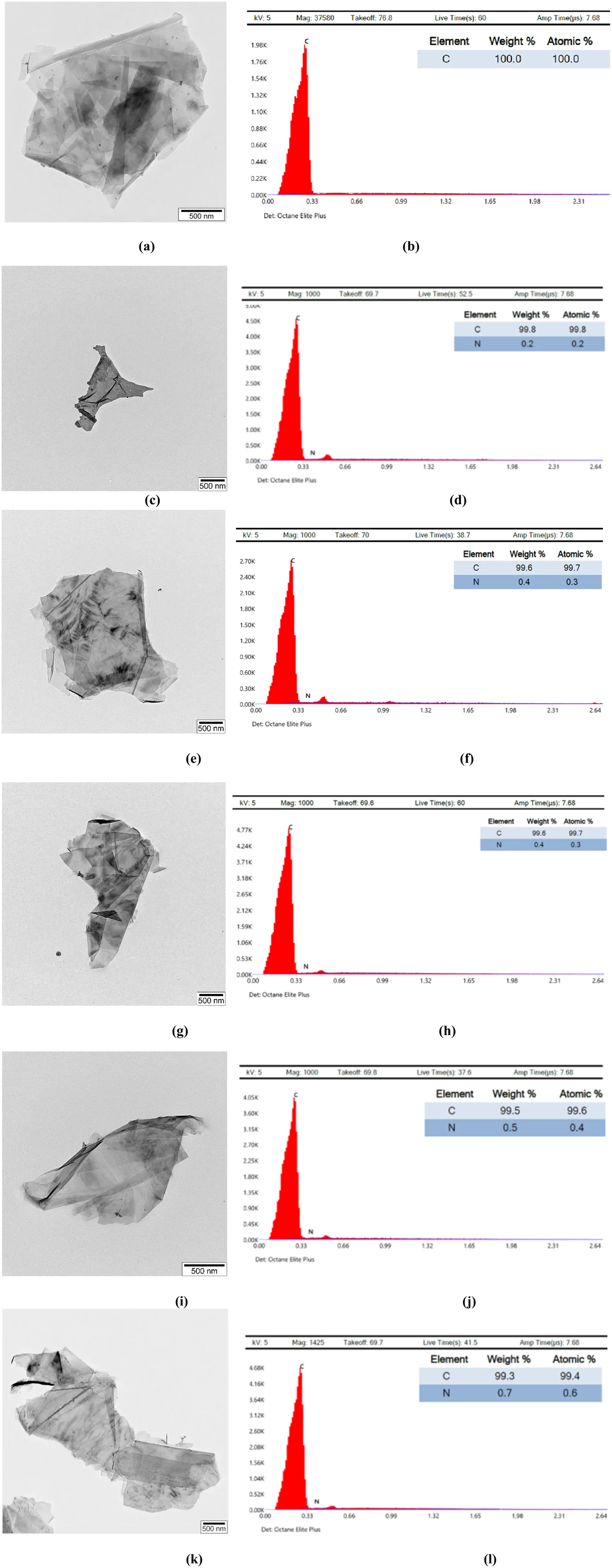
TEM images and EDX analysis of pure GNPs and N-functionalized GNPs samples: TEM: (a) GNPs; (c) N-GNPs 1%; (e) N-GNPs 2%; (g) N-GNPs 3%; (i) N-GNPs 4%; (k) N-GNPs 5%; EDX: (b) GNPs; (d) N-GNPs 1%; (f) N-GNPs 2%; (h) N-GNPs 3%; (j) N-GNPs 4%; (l) N-GNPs 5%.

The TEM images of pure GNPs (Fig. 5a) consist of thin, transparent, and large-sized graphene layers. The multilayer structure is clearly visible. Wrinkles and folds are observed at the edges, which are characteristic of exfoliated GNPs. Thick and dark areas indicate the formed multilayers. After adding a small amount – N-GNPs 1% (Fig. 5c), the size of the platelets decreases slightly and takes on a more irregular shape. The number of folds and folds at the edges of the layers increases. These changes can be attributed to local stresses resulting from surface functionalization. For N-GNPs 2% (Fig. 5e), the layers appear more wrinkled and more deformed. The appearance of different contrast areas indicates that the layers overlap in different directions. The maximum structural disorder in this sample is in good agreement with the highest *I*_D_/*I*_G_ ratio observed in the Raman results. For N-GNPs 3% (Fig. 5g), the layers again appear relatively compact. Signs of local restacking are observed. This situation is consistent with the decrease in interlayer spacing in XRD and the decrease in *I*_D_/*I*_G_ in Raman. For N-GNPs 4% (Fig. 5i), the large, thin, and translucent layers again dominate. The integrity of the layers is preserved, but wrinkles are still present at the edges. This can be explained by the more stable arrangement of N atoms within the structure and the occurrence of a partial π–π restacking process. The TEM image of the 5% N-GNPs (Fig. 5k) sample reveals relatively large, multilayer graphene sheets with pronounced overlapping and folded regions. Compared with the lower N concentrations, the sheets appear more compact and exhibit darker contrast in several areas, indicating increased local stacking of graphene layers. Despite this partial restacking, the graphene sheets remain thin and continuous without obvious fragmentation, demonstrating that the graphitic framework is preserved even at the highest N concentration. The observed morphology suggests that higher N loading promotes stronger intersheet interactions and localized π–π restacking while maintaining the overall layered structure of graphene.

For pure GNPs (Fig. 5b), the atomic and weight percentages of C were both 100%. For N-GNPs 1% (Fig. 5d), the atomic and weight % of C and nitrogen (N) were 99.8% and 0.2%, respectively. For N-GNPs 2% (Fig. 5f), the weight % of C and N were 99.6% and 0.4%, respectively, while the atomic % were 99.7% and 0.3%, respectively. For N-GNPs 3% (Fig. 5h), the weight % of C and N were 99.6% and 0.4%, respectively, while their atomic % were 99.7% and 0.3%, respectively. For N-GNPs 4% (Fig. 5j), the weight % of C and N were 99.5% and 0.5%, respectively, while the atomic % were 99.6% and 0.4%, respectively. Finally, for N-GNPs 5% (Fig. 5l), the weight % of C and N were 99.3% and 0.7%, respectively, while the atomic % were 99.4% and 0.6%, respectively. The sequential increase in both atomic % and weight % values, in line with the nominal values, confirms their successful incorporation into the GNP structure. It appears that no additional elements were detected as residues.

### 3.2. X-ray diffraction analysis

The XRD patterns of pure GNP and N-GNPs samples are shown in Fig. 6. All samples display the main graphitic (002) reflection near 2*θ* = 26.3–26.5°, together with weaker reflections assigned to higher-order graphitic planes.^40^ The persistence of these reflections after treatment indicates that the multilayer graphitic framework is preserved. No strong new crystalline phase is detected in the XRD patterns, suggesting that the dopamine/HCl treatment does not generate a separate crystalline impurity phase at a level detectable by XRD.

**Fig. 6:**
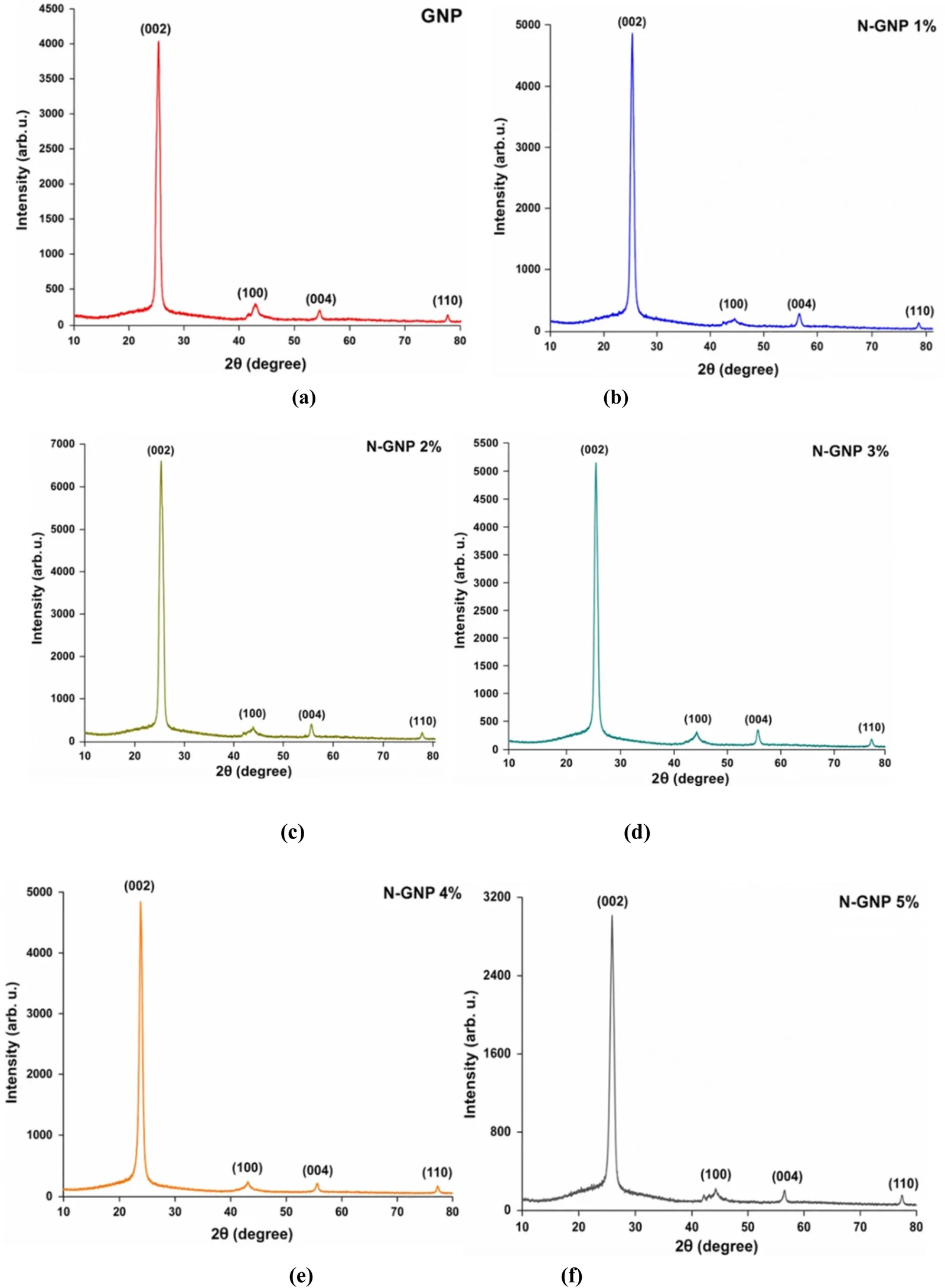
XRD patterns of pure GNPs and N-functionalized GNPs samples: (a) GNPs, (b) N-GNPs 1%, (c) N-GNPs 2%, (d) N-GNPs 3%, (e) N-GNPs 4%, and (f) N-GNPs 5%.

A non-monotonic change is observed in the (002) diffraction peak of GNPs after N modification. In the N-GNPs 1% and 2% samples, the peak shifts to smaller 2θ angles (26.249° and 26.269°), resulting in an increase in the interlayer spacing (*d*_002_) from 0.33724 nm to 0.33924 and 0.33898 nm, respectively.^41,42^

The fundamental relationship between the X-ray diffraction angle (°) and the interlayer spacing (*d*) is quantitatively governed by Bragg's law (eqn (2)):^43^

2 *λ* = 2*d *sin* θ*

where *λ* is the X-ray wavelength (for the standard Cu–Kα radiation source), *λ* = 0.15406 nm, *d* represents the interlayer spacing, and *θ* is the Bragg diffraction angle. This indicates that the interlayer spacing is slightly widened due to the formation of N-containing functional groups and local structural distortions. At low dopamine concentrations, N-containing functional groups introduced at platelet edges slightly increase the average interlayer spacing because of local steric effects. When the dopamine concentration increases to 3–5%, the (002) peak shifts again to larger angles and *d*_002_ decreases to 0.33806–0.33576 nm, which can be attributed to a closer stacking of layers (π–π restacking). This behavior suggests that the initial interlayer expansion is progressively overcome by microwave-induced π–π restacking, promoted by the partial removal or rearrangement of intercalated species and O-containing functional groups, leading to a more compact multilayer graphene structure.

The full width at half maximum (FWHM) of the (002) peak decreases from 0.599° in pure GNP to approximately 0.498–0.512° in the N-GNPs samples. The consistently lower FWHM values compared to pure GNPs indicate that N-functionalized graphene does not disrupt the graphitic framework. Instead, it introduces local defects while preserving the long-range stacking order, consistent with the observed non-monotonic evolution of *d*_002_.

These results suggest that the structural evolution is governed by two competing mechanisms: local expansion of the graphene layers due to N-containing functional groups at low dopamine concentrations, and progressive layer restacking induced by enhanced π–π interactions at higher dopamine concentrations. This interpretation aligns with earlier thermal and electrical studies of related GNPs, where packing and interflake contact geometry are central to macroscopic behavior.^28,30,31^ XRD parameters calculated from the (002) reflection of pure GNPs and N-GNPs samples are shown in Table 2.

**Table 2:** XRD parameters calculated from the (002) reflection of pure GNP and N-GNP samples

Sample	2*θ* (°)	*d*_002_ (nm)	FWHM (°)	Main interpretation
GNPs	26.408(7)	0.33724(9)	0.599(6)	Baseline multilayer graphitic stacking
N-GNPs 1%	26.249(8)	0.33924(10)	0.509(7)	Small expansion of interlayer spacing
N-GNPs 2%	26.269(4)	0.33898(5)	0.504(3)	Small expansion; framework preserved
N-GNPs 3%	26.342(3)	0.33806(4)	0.512(3)	Close to pure spacing
N-GNPs 4%	26.456(3)	0.33664(4)	0.498(3)	Slight contraction/restacking possible
N-GNPs 5%	26.526(4)	0.33576(5)	0.507(4)	Slight contraction; no collapse of graphitic structure

### 3.3. FTIR analysis

FTIR spectroscopy was used to evaluate changes in local bonding after N modification of GNPs. Fig. 7 demonstrates FTIR spectra of pure GNPs, as well as N-functionalized GNPs samples.

**Fig. 7:**
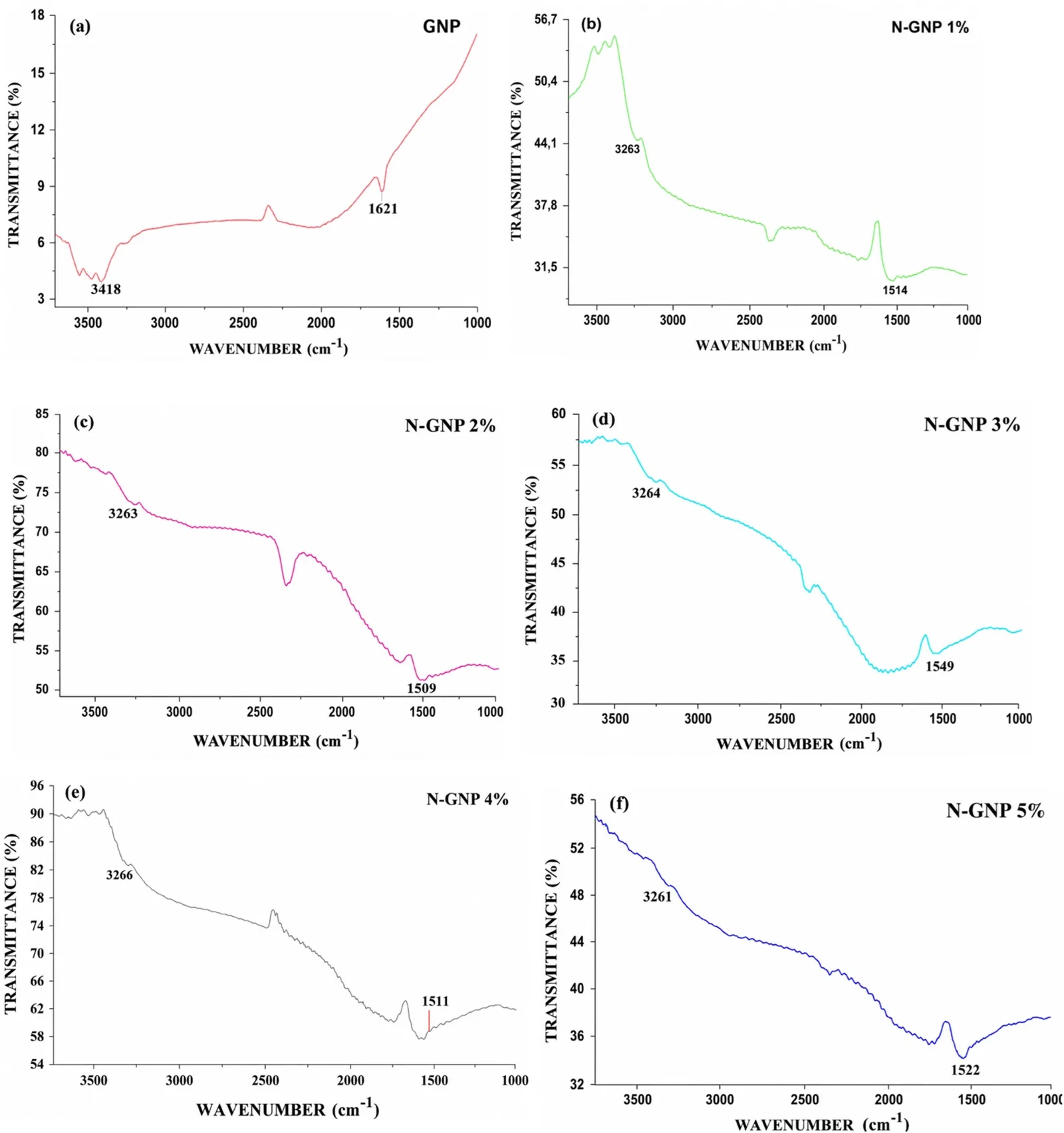
FTIR spectra of pure GNPs and N-functionalized GNP samples: (a) GNPs, (b) N-GNPs 1%, (c) N-GNPs 2%, (d) N-GNPs 3%, (e) N-GNPs 4%, and (f) N-GNPs 5%.

The pure GNPs spectrum (Fig. 7a) contains a band near 1621 cm^−1^, assigned mainly to skeletal C=C vibrations of the sp^2^ carbon framework.^44^ A broad band near 3418 cm^−1^ is attributed mainly to absorbed water and surface O–H groups.^45^ The absence of strong bands in the 1720–1740 cm^−1^ region and the weak response in the 1000–1250 cm^−1^ region indicate that the starting GNPs are not strongly oxidized and do not resemble highly oxidized graphene oxide. This is highly characteristic of pure graphitic nanomaterials, which typically exhibit negligible or very weak O-containing functional group bands in contrast to their heavily oxidized counterparts.^46,47^

After modification, the main C skeletal band shifts into the 1509–1549 cm^−1^ region.^48^ This shift supports perturbation of the sp^2^ carbon network and the contribution of C=N/C–N-related vibrations.^49^ The emergence or strengthening of features near 1000–1250 cm^−1^ is consistent with C–N stretching modes, while the broad band around 3261–3266 cm^−1^ can include contributions from O–H and N–H groups.^50^ These changes are consistent with N-containing surface or edge functionalities derived from dopamine treatment.

The evolution is non-monotonic. The concentration-dependent shift of the main FTIR band reflects the evolution of the local bonding environment induced by dopamine modification. At low dopamine concentrations (1–2%), the pronounced red shift indicates stronger perturbation of the sp^2^ C framework due to the introduction of N-containing functional groups, resulting in increased contributions from C=N/C–N vibrations and local changes in bond strength. At intermediate concentration (3%), the band shifts toward higher wavenumbers, suggesting partial restoration of the sp^2^ framework and a different balance between functional groups, local strain, and bond rigidity. With further increase in dopamine concentration (4–5%), the band shifts again toward lower/intermediate wavenumbers, indicating continued rearrangement of the local bonding environment. The non-monotonic behavior demonstrates that the FTIR band position is governed not simply by N concentration, but by the competition between N functionalization, local structural distortion, and changes in the electronic environment of the sp^2^ carbon network.

This FTIR interpretation is consistent with the XRD results. In the 1–2% N-GNPs samples, the strong red shift of the main FTIR band indicates pronounced perturbation of the sp^2^ carbon network by N-containing groups, while XRD shows a shift of the (002) peak to lower 2*θ* angles and an increase in *d*_002_, confirming slight interlayer expansion. In contrast, for the 3–5% samples, the FTIR band position changes toward higher/intermediate wavenumbers, suggesting rearrangement of the local bonding environment, whereas XRD shows a shift of the (002) peak to higher 2*θ* angles and a decrease in *d*_002_, indicating more compact layer stacking. Therefore, FTIR and XRD jointly show that dopamine-derived treatment produces a concentration-dependent competition between local functionalization/structural distortion and π–π restacking of graphene layers.

The FTIR results indicate concentration-dependent changes in the local bonding environment induced by dopamine modification and support the formation of N-containing surface functionalities.^5,15,16,51–53^ In Table 3, the main FTIR properties are shown.

**Table 3:** Main FTIR features and conservative structural interpretation

Sample	Main C=C/C=N band (cm^−1^)	Broad O–H/N–H band (cm^−1^)	Conservative interpretation
GNPs	1621	3418	sp^2^ carbon framework; adsorbed water/O–H; no strong oxidation signal
N-GNPs 1%	1514	3263	Strong local perturbation; C–N/C=N and N–H/O–H contributions likely
N-GNPs 2%	1509	3263	Highest red-shifted response; increased N-related functionalization
N-GNPs 3%	1549	3264	Partial recovery of skeletal-band frequency; different local bonding balance
N-GNPs 4%	1511	3266	Renewed low-frequency shift; stronger disorder/functionalization possible
N-GNPs 5%	1522	3261	Modified but partially stabilized local bonding response

### 3.4. Raman analysis

Raman spectroscopy is the most informative technique in the present dataset because it probes both graphitic order and defect activation. The Raman spectra of pure GNPs and N-functionalized GNPs are shown in Fig. 8.

**Fig. 8:**
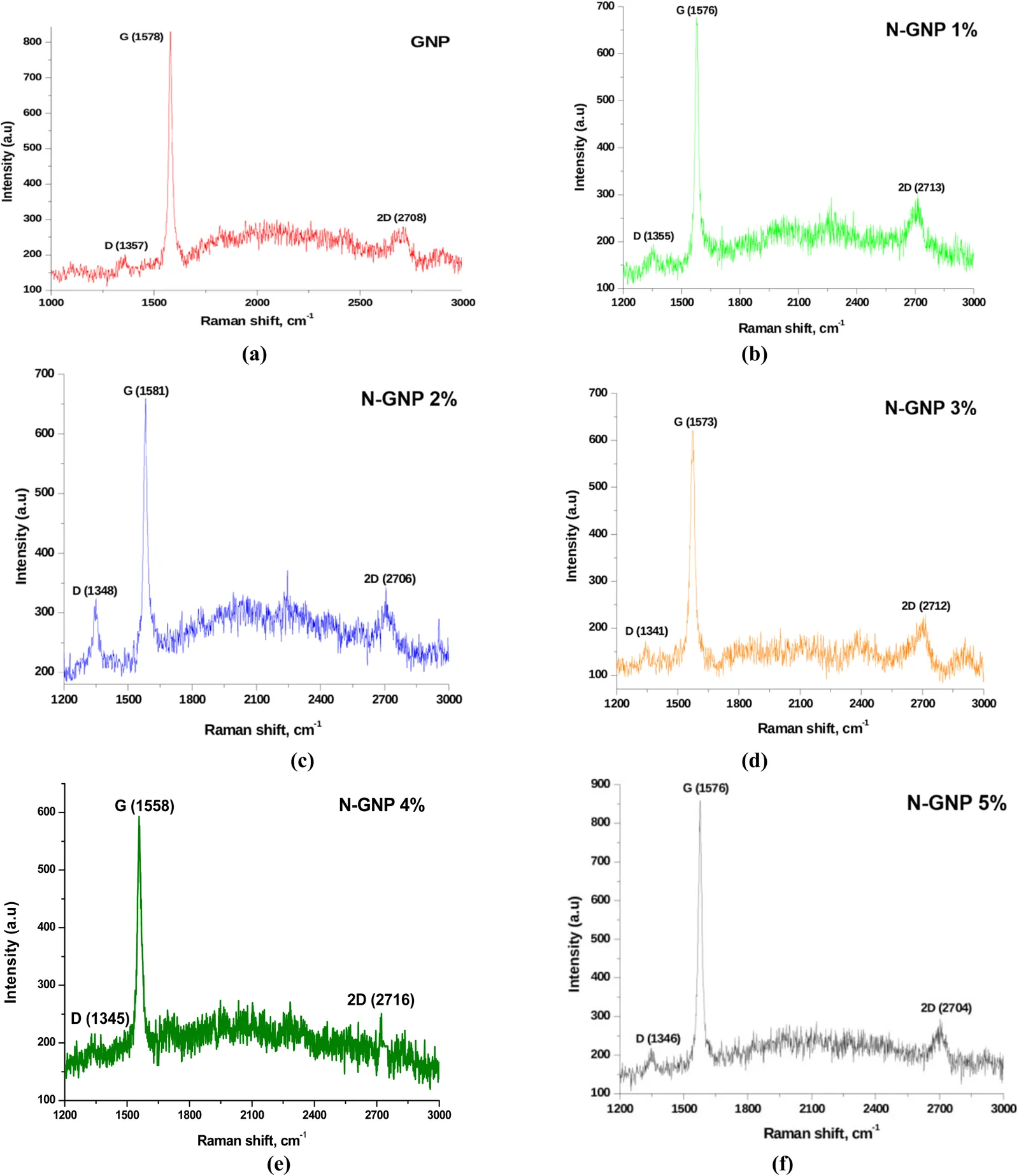
Raman spectra of pure GNPs and N-functionalized GNPs samples: (a) GNPs, (b) N-GNPs 1%, (c) N-GNPs 2%, (d) N-GNPs 3%, (e) N-GNPs 4% and (f) N-GNPs 5%.

For all samples, the spectra show the characteristic D, G, and 2D bands. The G band is associated with in-plane sp^2^ C vibrations, the D band is activated by defects, edges, and finite crystallite size, and the 2D band is sensitive to electronic structure, stacking, and layer number.^17–23^

Pure GNPs (Fig. 8a) show a G band at 1579 cm^−1^, a D band at 1357 cm^−1^, and a 2D band at 2708 cm^−1^.^54–56^ The *I*_D_/*I*_G_ ratio of 0.243 indicates a relatively low defect response for a nanoplatelet material, while the *I*_2D_/*I*_G_ ratio of 0.337 confirms a multilayer character rather than monolayer graphene.^57–59^

N modification changes both the peak positions (D band and G band) and intensity ratios, demonstrating modification of the vibrational and electronic structure. The *I*_D_/*I*_G_ ratio increases from 0.243 for pure GNPs to 0.289 for N-GNPs 1% (Fig. 8b) and reaches its maximum value of 0.492 for N-GNPs 2% (Fig. 8c). This indicates that the 2% modification produces the strongest activation of defect-related Raman scattering. The ratio then decreases to 0.281 for 3% (Fig. 8d), increases again to 0.363 for 4% (Fig. 8e), and falls close to the pure value, 0.253 at 5% (Fig. 8f). Therefore, the defect evolution is not linear with nominal N content. The non-monotonic trend may arise from competing processes: creation of C–N related defects, edge functionalization, changes in restacking, local strain, partial removal or rearrangement of unstable functional groups, and sample heterogeneity. This behavior is consistent with the XRD results. The increase in *d*_002_ for the 1–2% samples indicates slight interlayer expansion caused by N functionalization, whereas the simultaneous increase in *I*_D_/*I*_G_ reflects the creation of additional Raman-active defects. At higher dopamine concentrations (3–5%), the decrease in *d*_002_ together with the lower *I*_D_/*I*_G_ ratio suggests progressive layer restacking and structural reorganization rather than continuous defect generation. Compared with pure GNPs, all N-functionalized samples exhibit changes in both Raman peak positions and intensity ratios, confirming that dopamine changes the local vibrational and electronic environment of the sp^2^ C framework. The increase in the *I*_D_/*I*_G_ ratio from 0.243 (GNPs) to 0.289 (N-GNPs 1%) and 0.492 (N-GNPs 2%) indicates that N-containing functional groups are preferentially introduced at defect and edge sites, creating additional Raman-active defects.

The Raman results also agree with the FTIR analysis, where the largest red shift of the main skeletal vibration was observed for the 1–2% samples, indicating the strongest perturbation of the sp^2^ C framework. Both techniques, therefore, identify intermediate dopamine concentrations as producing the greatest local structural modification.

N incorporation into the graphene lattice is governed by the combined effects of atomic size, electronegativity, valence electron configuration, bond strength, and defect formation energy rather than by a single atomic parameter. N has a smaller covalent radius (56 pm) than C (67 pm), allowing substitutional incorporation into the graphene lattice with relatively small elastic distortion and better preservation of the hexagonal sp^2^ framework.^60^ Structurally, pure graphene possesses an sp^2^ C–C bond length of approximately 1.42 Å, whereas the C–N bond formed after substitutional N modification is typically 1.37–1.41 Å, depending on the N configuration (graphitic, pyridinic, or pyrrolic). The slightly shorter C–N bond originates from the smaller atomic radius of N and contributes to maintaining the structural integrity of the graphene lattice without significant lattice expansion.^61^ N is also more electronegative than C (*χ*_N_ = 3.04–3.05; *χ*_C_ = 2.55), resulting in polarization of the C–N bond and redistribution of electron density from neighboring C atoms toward the N site. This local redistribution perturbs the electronic structure and modifies the density of electronic states near the Fermi level.^62^

The apparent in-plane crystallite size (*L*_a_) was estimated using the Cançado relation.^20^ For the 532 nm excitation wavelength employed in this study. These values should be treated as apparent Raman domain sizes rather than absolute physical grain sizes, especially for chemically functionalized multilayer nanoplatelets. The general empirical equation establishing the relationship between the domain sizes and the laser excitation wavelength is expressed as eqn (3):3
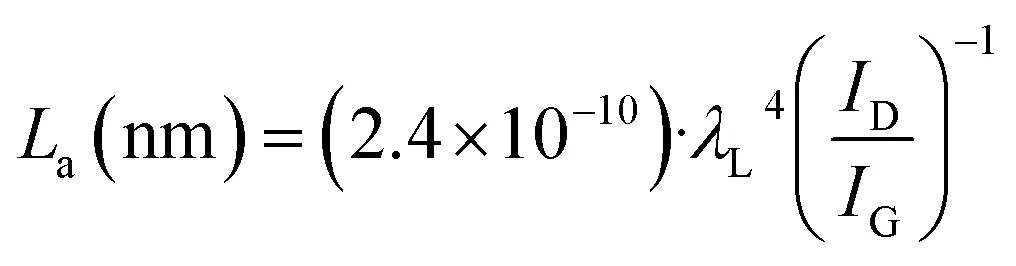
where *λ* is the laser excitation wavelength (nm), and *I*_D_/*I*_G_ is the intensity ratio of the D and G Raman bands. Since a 532 nm excitation laser was employed in this study, substituting *λ* = 532 nm into the general equation yields the following simplified expression (eqn (4)):4
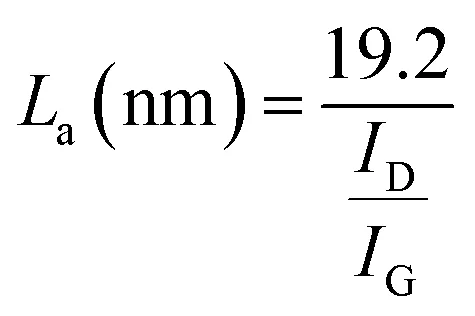


The smallest apparent *L*_a_ is observed for the 2% sample, consistent with its highest *I*_D_/*I*_G_ ratio. The apparent increase in *L*_a_ for N-GNPs 5% is consistent with the XRD evidence of reduced interlayer spacing, suggesting that enhanced π–π restacking reduces the Raman-active defect response without implying a complete recovery of the pure graphitic structure.

Raman parameters of pure and N-functionalized GNPs samples are given in Table 4.

**Table 4:** Raman parameters of pure and N-functionalized GNP samples. *L*_a_ was recalculated using *L*_a_(nm) approximately 19.2/(*I*_D_/*I*_G_) for 532 nm excitation

Sample	G (cm^−1^)	*I*_G_	D (cm^−1^)	*I*_D_	2D (cm^−1^)	*I*_2D_	*I*_D_/*I*_G_	*I*_2D_/*I*_G_	*L*_a_ approx. (nm)
GNPs	1579	832	1357	203	2708	281	0.243	0.337	79.1
N-GNPs 1%	1576	678	1355	196	2713	300	0.289	0.442	66.5
N-GNPs 2%	1581	660	1348	325	2706	343	0.492	0.519	39.1
N-GNPs 3%	1573	621	1341	175	2712	231	0.281	0.371	68.4
N-GNPs 4%	1558	594	1345	216	2716	252	0.363	0.424	53.0
N-GNPs 5%	1576	861	1346	218	2704	295	0.253	0.342	76.0

The N-containing defects can improve charge–transfer reactions and electrochemical response in selected systems, but the active site depends on the N configuration and on the surrounding C network.^4,24–26^ In GNP contacts, conductivity and permittivity depend on both the material and the contact network.^28^ N-functionalization may increase carrier density or alter local electronic structure, but it may also modify interflake junctions and surface barriers.

### 3.5. XPS analysis

XPS measurements were performed to investigate the chemical nature of the N modification of GNPs. The results are presented in Fig. 9. The collected survey spectra of all studied materials showed C, O, N, and Cl signals originating from GNPs, as well as Au from the substrate. The observed variations in the Au/C ratio reflect the differences in the local powder coverage and exposed substrate area during XPS analysis.

**Fig. 9:**
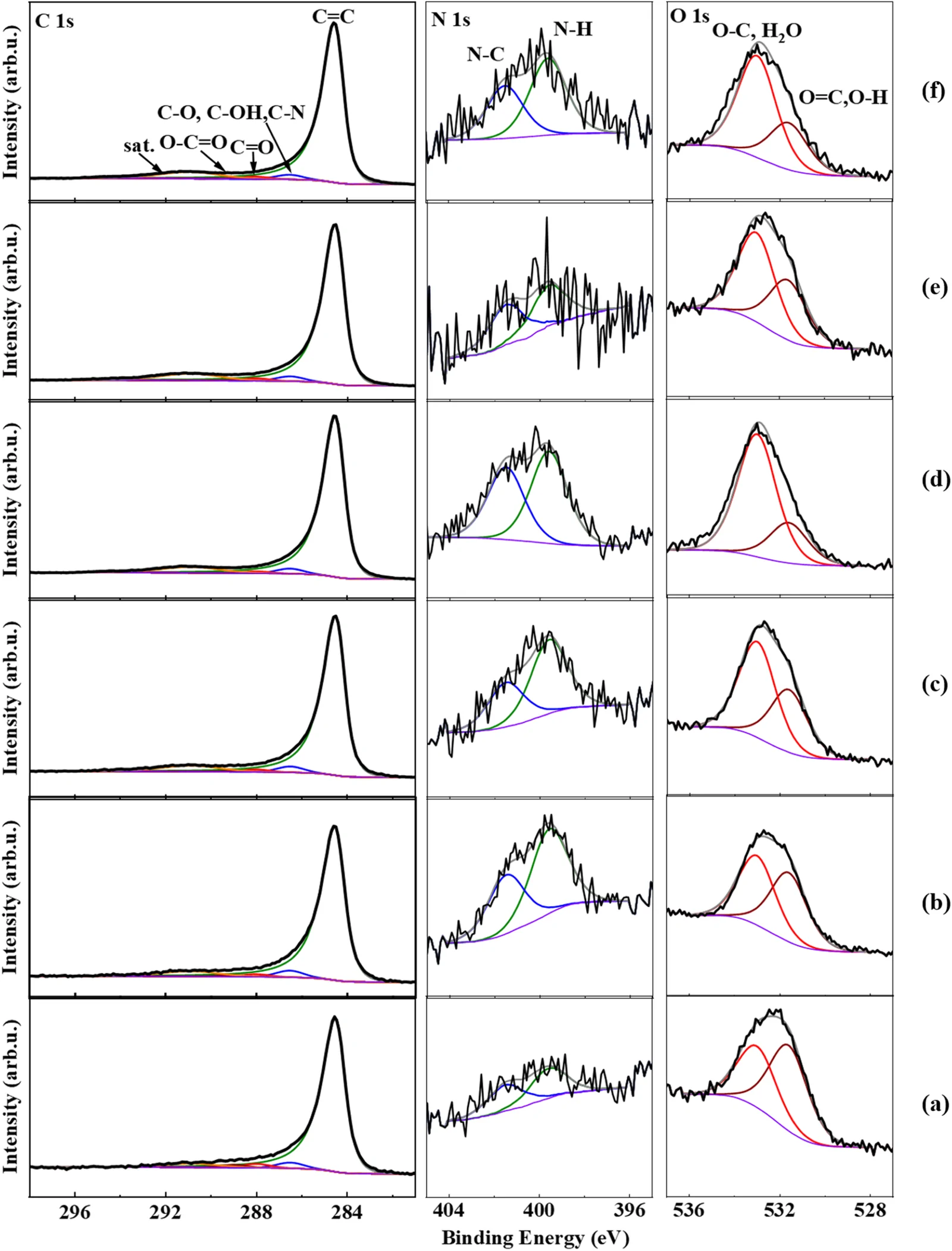
Normalized C 1s, N 1s, and O 1s XPS spectra of (a) GNPs, (b) N-GNPs 1%, (c) N-GNPs 2%, (d) N-GNPs 3%, (e) N-GNPs 4%, and (f) N-GNPs 5%.

The relevant compositional trends are discussed in terms of O/C, N/C, and Cl/C ratios (Table 5), while recognizing that the local powder coverage can influence signal intensity and quantification uncertainty. A weak N signal is already detected for pure GNPs (N/C = 0.007). This signal may originate from trace surface contamination or adventitious N-containing species and is therefore treated as a background/baseline contribution rather than evidence of intentional N modification. Relative to this baseline, the N/C ratio increases to 0.014 and 0.009 for N-GNPs 1% and 2%, respectively, whereas values of 0.007, 0.001, and 0.003 are obtained for N-GNPs 3%, 4%, and 5%, respectively. Thus, the N/C ratio does not increase monotonically with nominal dopamine concentration. The XPS results provide the clearest evidence for enhanced surface N in the 1% and 2% samples, while the N/C ratios of the higher-concentration samples are comparable to and lower than those of pure GNPs.

**Table 5:** XPS elemental ratios with respect to carbon and fitted component assignment for pure and N-functionalized GNP samples

Sample	C	O/C	N/C	Cl/C	Au/C
GNPs	1	0.073	0.007	0.009	0.35
	284.5 eV – 89%	531.6 eV – 58%	399.6 eV – 53%	197.5 eV	84.0 eV
	286.5 eV – 3%				
	288.0 eV – 2%	533.0 eV – 42%	401.5 eV – 47%		
	289.0 eV – 2%				
	290.9 eV – 4%				
N-GNPs 1%	1	0.063	0.014	0.018	0.28
	284.5 eV – 89%	531.6 eV – 50%	399.6 eV – 58%	197.4 eV	84.0 eV
	286.5 eV – 3%	533.0 eV – 50%	401.5 eV – 42%		
	288.0 eV – 1%				
	289.0 eV – 1%				
	290.9 eV – 6%				
N-GNPs 2%	1	0.038	0.009	0.006	0.10
	284.5 eV – 88%	531.6 eV – 36%	399.6 eV – 63%	197.8 eV	84.0 eV
	286.5 eV – 3%	533.0 eV – 64%	401.5 eV – 37%		
	288.0 eV – 1%				
	289.0 eV – 1%				
	290.9 eV – 7%				
N-GNPs 3%	1	0.029	0.007	0.002	0.004
	284.5 eV– 89%	531.6 eV – 25%	399.6 eV – 56%	198.4 eV	84.0 eV
	286.5 eV– 3%	533.0 eV – 75%	401.5 eV – 44%		
	288.0 eV – 1%				
	289.0 eV – 0%				
	290.9 eV – 7%				
N-GNPs 4%	1	0.02	0.001	0.001	0.03
	284.5 eV – 88%	531.6 eV – 40%	399.6 eV – 50%	197.8 eV	84.0 eV
	286.5 eV – 3%	533.0 eV – 60%	401.5 eV – 50%		
	288.0 eV – 1%				
	289.0 eV – 0%				
	290.9 eV – 8%				
N-GNPs 5%	1	0.02	0.003	0	0.001
	284.5 eV – 88%	531.6 eV – 32%	399.6 eV – 59%	—	84.0 eV
	286.5 eV – 3%	533.0 eV – 68%	401.5 eV – 41%		
	288.0 eV – 1%				
	289.0 eV – 0%				
	290.9 eV – 8%				

The C 1s spectra are dominated by the graphitic sp^2^ C=C component at 284.5 eV, which accounts for 88–89% of the fitted C 1s envelope. Minor components centered at approximately 286.5, 288.0, and 289.0 eV are assigned to C–OH/C–O–C/C–N, C=O, and O–C=O contributions. The feature at 290.9 eV is assigned to the π–π* shake-up satellite, following established XPS assignments for graphitic carbon materials.^63^ The absence of a distinct sp^3^ C–C/C–H component near 285.0 eV supports the conclusion that the graphitic framework remains largely preserved after the HCl/dopamine treatment.

The N 1s spectra were fitted with two components located at 399.6 and 401.5 eV. The component at ∼399.6 eV is attributed predominantly to pyrrolic/amine-type N environments, which can be associated with cyclized and residual dopamine-derived species. The higher-binding-energy component at ∼401.5 eV may include contributions from graphitic N as well as protonated amine species associated with dopamine-derived surface functionalities.^13^ Because these N environments can occur within overlapping N 1s binding-energy ranges, an unequivocal assignment to a single N configuration based solely on XPS is not possible. The relative contributions of the ∼399.6 and ∼401.5 eV components are approximately 50–63% and 37–50%, respectively. Thus, the N 1s spectra indicate the presence of different N-containing chemical environments associated with the HCl/dopamine treatment, while individual N configurations should be interpreted cautiously.

The fitted O 1s components at 531.6 and 533.0 eV are assigned to O=C/OH and O–C/adsorbed water, respectively.^64^ Notably, the O/C ratio decreases from 0.073 for pristine GNPs to 0.020 for N-GNPs 4% and 5%, indicating a decrease in O-containing surface species after the treatment. This trend is consistent with the XRD results, where the decrease in *d*_002_ at higher nominal dopamine concentrations suggests closer layer stacking. The Cl 2p_3/2_ peak near 198 eV indicates residual chloride-containing species associated with the HCl/dopamine treatment.^65^

Interestingly, the maximum Raman *I*_D_/*I*_G_ ratio is observed for N-GNPs 2%, whereas the highest XPS N/C ratio occurs for N-GNPs 1%, demonstrating that Raman-active defect density and surface N concentration do not follow the same concentration dependence. The decrease in N/C at higher nominal dopamine concentrations may partly be associated with the increased restacking indicated by XRD, which can reduce the fraction of N-containing sites accessible within the XPS sampling depth; however, differences in surface coverage and removal of weakly bound dopamine-derived species during washing may also contribute. The XPS interpretation is further supported by the FTIR spectra, which show C=N/C–N-related vibrational features associated with N-containing functionalities.

### 3.6. Thermal analysis

DSC and TGA analyses were performed to examine the thermal stability of the samples, and the results are presented in Fig. 10.

**Fig. 10:**
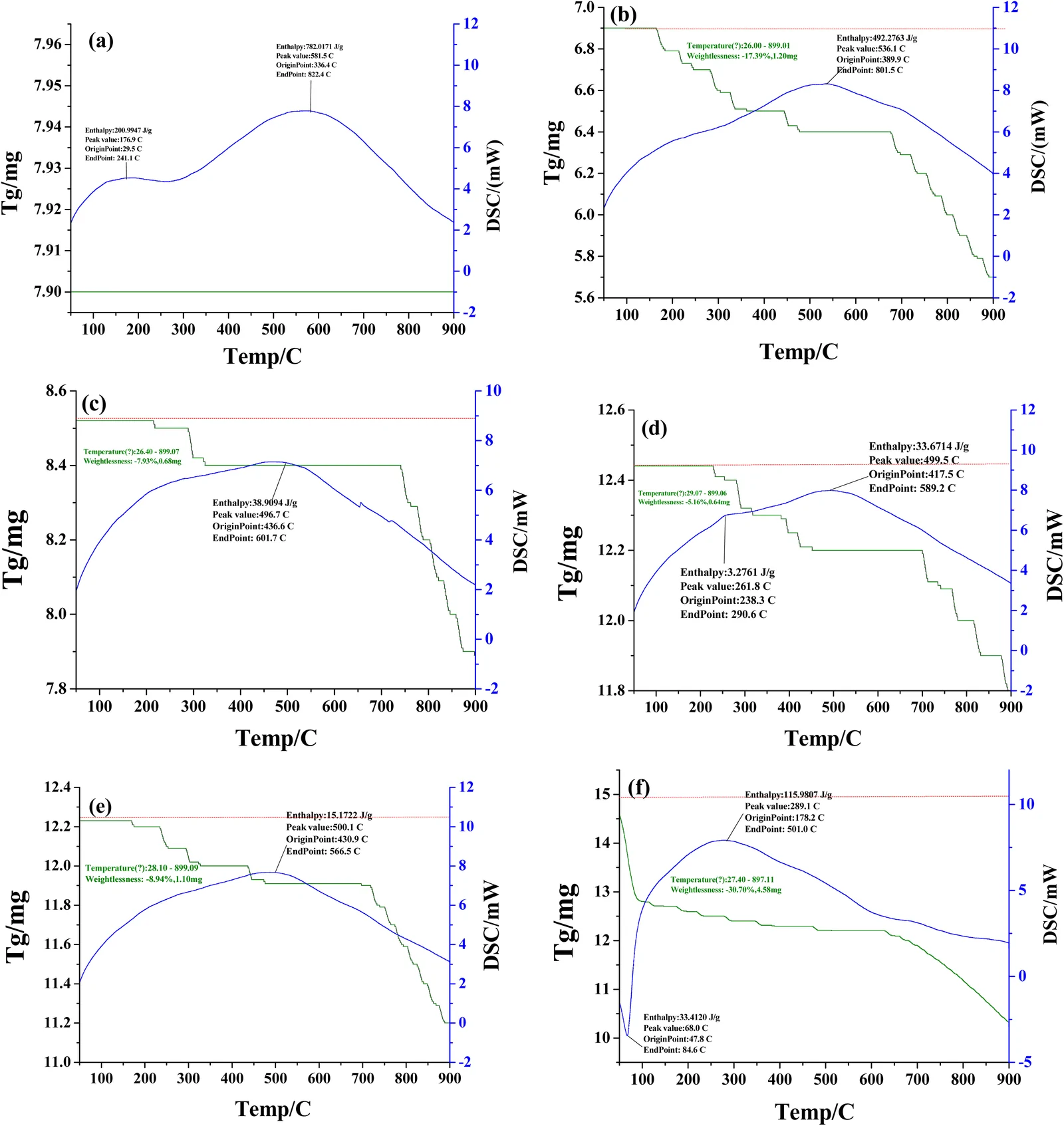
DSC analysis of pure GNPs (a), (b) N-GNPs 1%, (c) N-GNPs 2%, (d) N-GNPs 3%, (e) N-GNPs 4%, and (f) N-GNPs 5%.

The TGA curve of pure GNPs (Fig. 10a) remains essentially unchanged throughout the investigated temperature range, confirming mass loss up to 900 °C under N. This behavior demonstrates the high thermal stability of the graphitic carbon framework in an inert atmosphere. The DSC curve shows 2 broad peaks, including a weak event near 177 °C and a second broad thermal peak centered at approximately 582 °C. Since these properties are not accompanied by measurable mass loss, they are more likely related to thermally induced changes in the surface state and structural rearrangement of the GNPs layers rather than decomposition of the C framework.

1% N-GNPs show (Fig. 10b) a pronounced stepwise decrease in mass, from approximately 6.9 to 5.7 mg, corresponding to a total mass loss of about 17%. The main decreases occur over several temperature intervals rather than as a single decomposition step, suggesting the presence of thermally unstable surface species with different bonding environments. The broad DSC curve centered at approximately 536 °C, with an enthalpy of about 492 J g^−1^, occurs within the same general temperature region in which progressive mass loss is observed. The combined TGA–DSC behavior therefore indicates substantial thermally induced transformations of the functionalized surface and defect-associated species.

The 2% N-GNPs sample (Fig. 10c) exhibits a considerably smaller mass loss than the 1% sample. Its mass decreases from approximately 8.5 to 7.9 mg by 900 °C, corresponding to about 7% total mass loss. The TGA curve remains relatively stable through much of the intermediate-temperature region, followed by a more noticeable decrease at higher temperatures. A broad DSC curve is observed around 500 °C with an enthalpy of approximately 346 J g^−1^. These results suggest that the sample retains a relatively stable C framework while N functionalization and the associated defect/surface structure introduce thermally responsive sites.

The 3% N-GNPs sample (Fig. 10d) shows the lowest overall mass loss among the N-functionalized samples, decreasing from approximately 12.44 to 11.89 mg, corresponding to about 4.4%. The mass loss occurs through several small steps, particularly at elevated temperatures, rather than through rapid degradation. The DSC curve displays a broad peak around 494 °C, while the reported enthalpy is approximately 3.28 J g^−1^. The limited mass change indicates that most of the C framework is preserved up to 900 °C, although surface and defect-associated species undergo thermally induced transformations.

For 4% N-GNPs (Fig. 10e), the TGA curve again shows a stepwise profile, with an overall mass loss of approximately 7.6%. Relatively small changes occur at low and intermediate temperatures, followed by a more pronounced decrease above approximately 700 °C. The DSC curve contains a broad thermal event centered near 504 °C with an enthalpy of about 42.9 J g^−1^. The simultaneous TGA and DSC responses indicate thermally activated transformations associated with the functionalized surface and defect structure, while most of the graphitic framework remains intact over the investigated temperature range.

The 5% N-GNPs sample (Fig. 10f) exhibits an overall mass loss of approximately 6.8%. Unlike some of the lower-N samples, much of the pronounced mass decrease occurs in the higher-temperature region, particularly above approximately 600–700 °C. The DSC curve shows a broad thermal response with a maximum around 508 °C and an enthalpy of approximately 37.1 J g^−1^. Thus, despite the presence of thermally responsive surface and defect-related sites, the relatively limited total mass loss indicates that the main C framework remains stable under N up to high temperatures.

The N functionalization clearly changes the thermal response of GNPs, but the effect is non-monotonic with nominal N concentration. Pure GNPs show essentially no mass loss, whereas the N-functionalized samples exhibit approximately 17%, 7%, 4.4%, 7.6%, and 6.8% mass loss for 1, 2, 3, 4, and 5% N-GNPs, respectively. Thus, increasing the nominal N content does not lead to a progressive decrease in thermal stability; instead, the 1% sample shows the greatest mass loss, while 3% N-GNPs show the lowest mass loss among the functionalized samples. The main broad DSC properties also shift from approximately 582 °C for pure GNP to 494–536 °C for the N-functionalized samples, demonstrating that functionalization alters the thermal properties of the GNPs structure. Taken together, these results suggest that thermal behavior is governed not simply by nominal N concentration, but by the resulting surface functionalities, defect structure, N bonding configurations, and structural organization of the GNPs layers.

### 3.7. BET surface-area analysis

BET analysis was used to evaluate whether N-modification alters the accessible surface area of the GNP network, and the results are presented in Table 6. Pure GNPs show a specific surface area of 29.8 m^2^ g^−1^. The treated samples show a response: 37.0 m^2^ g^−1^ for N-GNPs 1%, 45.6 m^2^ g^−1^ for N-GNPs 2%, 27.7 m^2^ g^−1^ for N-GNPs 3%, 37.6 m^2^ g^−1^ for N-GNPs 4%, and 32.0 m^2^ g^−1^ for N-GNPs 5%.

**Table 6:** BET specific surface area of pure and N-functionalized GNP samples

Sample	BET specific surface area (m^2^ g^−1^)
GNPs	29.8
N-GNPs 1%	37.0
N-GNPs 2%	45.6
N-GNPs 3%	27.7
N-GNPs 4%	37.6
N-GNPs 5%	32.0

The BET results reveal a non-monotonic evolution of the specific surface area following N modification. Compared to pure GNPs (29.8 m^2^ g^−1^), the specific surface area increases to 37.0 m^2^ g^−1^ for N-GNPs 1% and reaches a maximum of 45.6 m^2^ g^−1^ for N-GNPs 2%, indicating that moderate N incorporation effectively increases the accessible surface area. This increase can be attributed to the generation of structural defects, exposure of new edge sites, sheet corrugation, and partial exfoliation, all of which enhance the accessibility of N molecules during BET measurements. At higher N concentrations, the specific surface area decreases to 37.6 m^2^ g^−1^ for N-GNPs 4% and 32.0 m^2^ g^−1^ for N-GNPs 5%, suggesting that defect generation gradually competes with partial restacking and aggregation of graphene sheets, thereby reducing the accessible surface area. The lowest value observed for N-GNPs 3% (27.7 m^2^ g^−1^) further indicates that local aggregation or pore blocking can temporarily dominate over defect-induced surface development.

Considering the dependence of the specific surface area on the domain size, it may be determined from eqn (5).^66^5
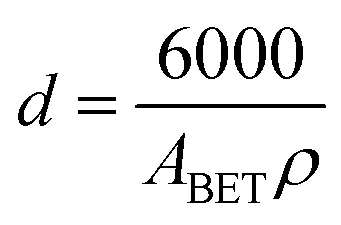
where *d* is the domain size, and *A*_BET_ is the specific surface area, and *ρ* is the theoretical density. BET analysis was performed to evaluate the influence of N modification on the accessible surface area of the GNPs. The pure GNPs exhibited a specific surface area of 29.8 m^2^ g^−1^. Following N modification, the surface area increased to 37.0 m^2^ g^−1^ for N-GNPs 1%, indicating that the introduction of structural defects, exposure of new edge sites, sheet corrugation, and partial exfoliation enhanced the accessibility of N_2_ molecules during BET measurements. The specific surface area then increased to 45.6 m^2^ g^−1^ for N-GNPs 2% and reached a minimum value of 27.7 m^2^ g^−1^ for N-GNPs 3%, suggesting that local aggregation and partial pore blocking temporarily became dominant over defect-induced surface development. Subsequently, the surface area increased to 37.6 m^2^ g^−1^ for N-GNPs 4% and reached its maximum value of 32.0 m^2^ g^−1^ for N-GNPs 5%, indicating that further structural reorganization restored the accessibility of the pore network. The minimum BET surface area observed for the 3% sample is consistent with its role as a structural transition point. At this concentration, the initial defect formation and interlayer expansion observed at lower dopamine concentrations are progressively replaced by local structural reorganization and partial layer restacking. This interpretation is supported by the simultaneous decrease in the interlayer spacing (*d*_002_) obtained from XRD, the shift of the characteristic FTIR bands toward higher wavenumbers, and the minimum BET specific surface area, indicating a transition from the functionalization stage to the structural reorganization/restacking stage.

The XPS results provide direct chemical evidence for this behavior by confirming the incorporation of N into the graphene framework through pyrrolic N (399.6 eV) and graphitic N (401.5 eV) species. These N configurations modify the local bonding environment, introduce lattice distortion and edge defects, and therefore contribute to the increase in accessible surface area observed at moderate N concentrations. This interpretation is further supported by Raman spectroscopy. The decrease in the Raman-derived lateral crystallite size (*L*_a_) from pure GNPs to N-GNPs 2% indicates progressive disruption of the ordered sp^2^ domains, consistent with the increase in the BET surface area. The subsequent increase in *L*_a_ for higher N concentrations, together with the decrease in BET surface area, suggests partial recovery of larger coherent graphitic domains accompanied by restacking and reduced accessibility of the graphene surface.

### 3.8. Electrical measurements

The electrical properties of pure and N-functionalized GNPs were evaluated to determine the effect of N content on charge transport, as summarized in Table 7.

**Table 7:** Electrical resistivity and conductivity of pure and N-functionalized GNPs

Samples	*ρ* (Ω cm)	*σ* (S cm^−1^)
GNPs	0.0113	88.496
N-GNPs 1%	0.0194	51.546
N-GNPs 2%	0.0138	72.727
N-GNPs 3%	0.459	2.179
N-GNPs 4%	0.02	50.0
N-GNP 5%	0.039	25.641

The electrical measurements showed a non-linear effect of N modification on the conductivity of GNPs. Pure GNP exhibited a conductivity of 88.496 S cm^−1^. After N modification, the conductivity decreased to 51.546 S cm^−1^ at N-GNPs 1% and then increased to 72.727 S cm^−1^ at N-GNPs 2%, which was the highest value among the N-functionalized samples. This indicates that at N-GNPs 2%, the conductive graphene network is still well preserved despite the structural changes introduced by N.

Importantly, the designation of N-GNPs 2% as the optimum composition is based on the balance among the measured properties rather than on electrical conductivity alone. Its conductivity (72.727 S cm^−1^) is the highest among the N-functionalized samples, although it remains lower than that of pure GNPs (88.496 S cm^−1^). At the same time, N-GNPs 2% exhibits the highest accessible BET surface area (45.6 m^2^ g^−1^) and the highest Raman *I*_D_/*I*_G_ ratio (0.492), indicating enhanced surface accessibility and defect-related sites while retaining comparatively efficient electrical transport. Thus, the 2% sample represents a favorable experimental balance between increased accessible surface area and preservation of conductive pathways rather than a maximum in conductivity itself.

A sharp decrease in conductivity was observed for N-GNPs 3% (2.179 S cm^−1^). The structural results suggest that partial restacking and reorganization of the graphene layers may contribute to this decrease; however, restacking should not be considered the sole origin of the reduced conductivity. In a macroscopic GNP network, electrical transport also depends on specimen packing, platelet orientation, and the number and quality of interflake contacts. Variations in these parameters can increase interflake contact resistance and disrupt continuous conductive pathways. Therefore, the low conductivity of N-GNPs 3% may reflect the combined effects of partial restacking, N-induced defects and carrier scattering, as well as differences in specimen packing and interflake connectivity.

At N-GNPs 4%, the conductivity partially recovered to 50.0 S cm^−1^, followed by a decrease to 25.641 S cm^−1^ at N-GNPs 5%. This non-monotonic behavior suggests that electrical transport is influenced by a combination of N-induced structural modification, defect formation, layer restacking, and network-level factors such as specimen packing and interflake connectivity. Among the N-functionalized samples, N-GNPs 2% therefore provides the most favorable balance of the measured properties, combining the highest accessible surface area with the highest conductivity within the treated series, although its conductivity remains below that of pristine GNPs.

### 3.9. Mechanistic interpretation of dopamine-derived N modification

A plausible mechanism begins with the adsorption of dopamine-derived species onto graphene surfaces, particularly at edge defects and residual O-containing groups, under acidic conditions (pH 4–5). During sonication and microwave treatment, dopamine undergoes oxidation and partial decomposition, generating reactive N-containing species that predominantly functionalize edge and defect sites, with limited substitution of carbon atoms at energetically favorable defects.^4,10–14,24–26^ The subsequent drying step at 150 °C promotes local condensation but is insufficient for extensive substitution throughout the basal plane. This mechanism is consistent with experimental results. XPS confirms the presence of pyrrolic and graphitic N species, XRD indicates preservation of the graphitic framework, FTIR reveals the formation of N-containing functional groups, while Raman and BET demonstrate that N incorporation increases defect density and accessible surface area without destroying the overall multilayer graphene structure.

### 3.10. Comparison with broader N-functionalized graphene literature

The present results agree well with previous studies showing that N modification alters graphene through two complementary mechanisms: electronic perturbation of the sp^2^ network and the generation of chemically active edge and defect sites.^4,10–14,24–26^ Compared with high-temperature ammonia or CVD treatments, the dopamine/HCl-assisted microwave route is considerably milder, leading predominantly to N incorporation at edge and defect sites while preserving the graphitic framework.^7,15,16^ These complementary results consistently demonstrate that the dopamine-assisted treatment modifies mainly the surface and defect structure of multilayer graphene without extensive reconstruction of the basal plane.^20–23^

### 3.11. Integrated interpretation

The significance of N functionalization in this study is its ability to simultaneously tune surface chemistry, defect structure, accessible surface area, and electrical transport in GNPs without destroying their multilayer graphitic framework. The combined characterization results reveal a non-monotonic relationship between N functionalization, structural disorder, surface properties, and electrical transport. SEM confirms that the characteristic wrinkled and layered morphology of GNPs is preserved after modification, while TEM shows the strongest sheet deformation and wrinkling for N-GNPs 2%; at higher concentrations, increasing overlap and partial restacking are observed. EDX detects N in the functionalized samples, with the measured N content increasing from 0.2 at% at 1% to 0.6 at% at 5%. XRD supports this structural evolution: (*d*_002_) increases from 0.33724 nm for pure GNPs to 0.33924 and 0.33898 nm at 1 and 2%, indicating slight functionalization-induced interlayer expansion, whereas its decrease to 0.33576 nm at 5% suggests progressive π–π restacking. FTIR further supports N functionalization through C–N/C=N-related vibrations, with the strongest skeletal-band shift observed at 2% (1509 cm^−1^). Raman similarly identifies 2% as the most defect-activated composition, where *I*_D_/*I*_G_ increases from 0.243 to 0.492, and *L*_a_ decreases from 79.1 to 39.1 nm. The subsequent decrease in *I*_D_/*I*_G_ at higher concentrations is consistent with structural reorganization and partial restacking. XPS reveals N 1s components at ∼399.6 and ∼401.5 eV, associated predominantly with pyrrolic/amine-type N, as well as, N environments that may include graphitic N and protonated amine contributions, respectively. The non-monotonic N/C ratio indicates that the surface-accessible N does not increase proportionally with nominal precursor concentration. The dominant sp^2^ C=C component at 284.5 eV (∼88–89%) confirms preservation of the graphitic framework. BET also exhibits a non-monotonic trend, changing from 29.8 m^2^ g^−1^ for pure GNPs to 37.0, 45.6, 27.7, 37.6, and 32.0 m^2^ g^−1^ for 1–5%, respectively. Notably, N-GNPs 2% combines the highest defect response with the largest accessible surface area (45.6 m^2^ g^−1^).

These structural and surface changes are reflected in electrical transport. N-GNPs 2% shows the highest conductivity among the N-functionalized samples (72.73 S cm^−1^), compared with 88.50 S cm^−1^ for pure GNPs. Among the investigated compositions, N-GNPs 2% provides the most favorable balance between defect generation, accessible surface area, and electrical conductivity while preserving the multilayer graphitic framework. At low nominal concentrations (1–2%), N modification is accompanied by increased defect density and surface accessibility while conductive pathways remain largely preserved. At higher concentrations (3–5%), structural reorganization and partial restacking increasingly influence surface accessibility and interflake charge transport.^3–7,24–32,67,68^

Thus, N modification enables a concentration-dependent balance between defect-associated and accessible surface sites and electrical transport, rather than simply maximizing a single property. This tunability is particularly relevant for applications where surface interactions and efficient charge transport are simultaneously required.

### 3.12. Application-oriented implications

The combined XRD, FTIR, Raman, XPS, and BET results indicate that N modification successfully tailors the surface chemistry and defect structure of GNPs while preserving the multilayer graphitic framework. Such a combination of preserved electrical pathways, N-containing active sites, and increased accessible surface area is considered advantageous for applications such as electrochemical electrodes, sensors, catalysts, adsorbents, and conductive polymer composites, where both graphitic conductivity and surface functionality are important.^3–7,15,16,24–32,67–69^

## 4. Conclusions

N-functionalized GNPs were successfully synthesized by an HCl/dopamine-assisted microwave route, and their structural evolution was found to be strongly dependent on the nominal N precursor concentration. XRD analysis confirmed that the multilayer graphitic framework remained preserved after modification, with the (002) reflection retained at 26.25–26.53° and only a slight variation in the interlayer spacing from 0.33724 nm for pure GNPs to 0.33924 nm (1%) and 0.33576 nm (5%). This non-monotonic behavior indicates a competition between initial interlayer expansion caused by N-containing functional groups and subsequent π–π restacking at higher precursor concentrations. TEM images demonstrated that N modification altered the morphology through increased sheet deformation and local restacking without disrupting the fundamental layered graphitic structure. FTIR and XPS support the formation of N-containing functionalities associated with C–N/C=N-related bonding, and as well as pyrrolic, graphitic N and/or amine, and protonated amine species. The non-monotonic evolution of the N/C ratio, with the clearest enhancement in surface N observed at lower nominal dopamine concentrations, indicates that increasing dopamine concentration does not directly translate into increased surface N content. Raman spectroscopy revealed that N modification predominantly affects the local sp^2^ C network. The N-GNPs 2% sample exhibited the highest defect activation with an *I*_D_/*I*_G_ ratio of 0.492, accompanied by the smallest apparent graphitic domain size (39.1 nm) compared with 79.1 nm for pure GNPs. At higher precursor concentrations, the decrease in *I*_D_/*I*_G_ together with the increase in the apparent domain size suggests progressive structural reorganization and partial restacking rather than continuous defect generation. The persistence of *I*_2D_/*I*_G_ < 1 for all samples confirms that the multilayer GNP structure is maintained throughout the modification process. The BET results further support this structural evolution. The specific surface area increased from 29.8 m^2^ g^−1^ for pure GNPs to a maximum of 45.6 m^2^ g^−1^ for N-GNPs 2%, confirming that moderate N modification maximizes accessible edge sites and defect-related surface development. The subsequent decrease in surface area for higher precursor concentrations is attributed to partial platelet restacking and pore blocking. Based on electrical measurements, N-GNPs 2% exhibited the most favorable combination of structural and electrical properties. The combined characterization demonstrates that N-GNPs 2% exhibited the most favorable balance among the measured properties, combining the highest BET surface area (45.6 m^2^ g^−1^), the strongest Raman defect response (*I*_D_/*I*_G_ = 0.492), and the highest conductivity among the N-functionalized samples (72.73 S cm^−1^), although the latter remained below that of pure GNPs (88.50 S cm^−1^). The pronounced conductivity decrease at 3% may be associated with partial restacking and structural reorganization, but variations in specimen packing and interflake contacts may also contribute to the measured electrical response. The observed concentration-dependent structural evolution is governed by the competition between N functionalization, defect formation, and π–π restacking, providing an effective strategy for tailoring the physicochemical properties of GNPs for advanced C-based functional materials.

## Author contributions

Conceptualization, L. G.; methodology, L. G.; validation, L. G.; formal analysis, Kh. M., M. V. D., and M. L.; investigation, L. G. and R. H.; data curation, L. G. and S. H.; writing – original draft preparation, L. G.; writing – review & editing, L. G., A. P., A. G., M. L., A. K. and L. C.; supervision, S. B., and M. M. All authors have read and agreed to the published version of the manuscript.

## Conflicts of interest

The authors declare no competing interests.

## Data Availability

The data that support the findings of this study are available from the corresponding author upon reasonable request.

## References

[cit1] Novoselov K. S., Geim A. K., Morozov S. V., Jiang D. E., Zhang Y., Dubonos S. V., Grigorieva I. V., Firsov A. A. (2004). Electric field effect in atomically thin carbon films. Science.

[cit2] Geim A. K., Novoselov K. S. (2007). The rise of graphene. Nat. Mater..

[cit3] Gomez V., Guevara M., Tene T., Villamagua L., Usca G. T., Maldonado F., Tapia C., Cataldo A., Bellucci S., Caputi L. S. (2021). The liquid exfoliation of graphene in polar solvents. Appl. Surf. Sci..

[cit4] Wang H., Maiyalagan T., Wang X. (2012). Review on recent progress in nitrogen-doped graphene: Synthesis, characterization, and its potential applications. ACS Catal..

[cit5] Peng C., Zhang X. (2021). Chemical functionalization of graphene nanoplatelets with hydroxyl, amino, and carboxylic terminal groups. Chemistry.

[cit6] Fan X., Chang D. W., Chen X., Baek J.-B., Dai L. (2016). Functionalized graphene nanoplatelets from ball milling for energy applications. Curr. Opin. Chem. Eng..

[cit7] Chang D. W., Lee E. K., Park E. Y., Yu H., Choi H.-J., Jeon I.-Y., Sohn G.-J., Shin D., Park N., Oh J. H., Dai L., Baek J.-B. (2013). Nitrogen-doped graphene nanoplatelets from simple solution edge-functionalization for n-type field-effect transistors. J. Am. Chem. Soc..

[cit8] Fan A., Zhang Q., Wang Y. (2025). *et al.*, Recent advances in layer engineering and controllable fabrication of graphene, h-BN, and transition metal dichalcogenides. Sci. China Mater..

[cit9] Cheng X., Chen B., Zhang L. (2025). *et al.*, Exfoliated metal oxide ultrathin nanosheets with supported Pd nanoparticles enabling the one-pot reaction of phenol into caprolactam. Sci. China: Chem..

[cit10] Panchakarla L. S., Subrahmanyam K. S., Saha S. K., Govindaraj A., Krishnamurthy H. R., Waghmare U. V., Rao C. N. R. (2009). Synthesis, structure, and properties of boron- and nitrogen-doped graphene. Adv. Mater..

[cit11] Wang X., Li X., Zhang L., Yoon Y., Weber P. K., Wang H., Guo J., Dai H. (2009). N-doping of graphene through electrothermal reactions with ammonia. Science.

[cit12] Schiros T., Nordlund D., Pálová L., Prezzi D., Zhao L., Kim K. S., Wurstbauer U. (2012). *et al.*, Connecting dopant bond type with electronic structure in N-doped graphene. Nano Lett..

[cit13] Lazar P., Mach R., Otyepka M. (2019). Spectroscopic fingerprints of graphitic, pyrrolic, pyridinic, and chemisorbed nitrogen in N-doped graphene. J. Phys. Chem. C.

[cit14] Vesel A., Zaplotnik R., Primc G., Mozetič M. (2020). A review of strategies for the synthesis of N-doped graphene-like materials. Nanomaterials.

[cit15] Lee H., Dellatore S. M., Miller W. M., Messersmith P. B. (2007). Mussel-inspired surface chemistry for multifunctional coatings. Science.

[cit16] Lee H., Dellatore S. M., Miller W. M., Messersmith P. B. (2007). Mussel-inspired surface chemistry for multifunctional coatings. Science.

[cit17] Ferrari A. C., Meyer J. C., Scardaci V., Casiraghi C., Lazzeri M., Mauri F., Piscanec S., Jiang D., Novoselov K. S., Roth S., Geim A. K. (2006). Raman spectrum of graphene and graphene layers. Phys. Rev. Lett..

[cit18] Ferrari A. C., Basko D. M. (2013). Raman spectroscopy as a versatile tool for studying the properties of graphene. Nat. Nanotechnol..

[cit19] Tuinstra F., Koenig J. L. (1970). Raman spectrum of graphite. J. Chem. Phys..

[cit20] Cançado L. G., Takai K., Enoki T., Endo M., Kim Y. A., Mizusaki H., Jorio A., Coelho L. N., Magalhães-Paniago R., Pimenta M. A. (2006). General equation for the determination of the crystallite size La of nanographite by Raman spectroscopy. Appl. Phys. Lett..

[cit21] Lucchese M. M., Stavale F., Ferreira E. H. M., Vilani C., Moutinho M. V. O., Capaz R. B., Achete C. A., Jorio A. (2010). Quantifying ion-induced defects and Raman relaxation length in graphene. Carbon.

[cit22] Eckmann A., Felten A., Mishchenko A., Britnell L., Krupke R., Novoselov K. S., Casiraghi C. (2012). Probing the nature of defects in graphene by Raman spectroscopy. Nano Lett..

[cit23] Kaniyoor A., Ramaprabhu S. (2012). A Raman spectroscopic investigation of graphite oxide derived graphene. AIP Adv..

[cit24] Qu L., Liu Y., Baek J.-B., Dai L. (2010). Nitrogen-doped graphene as efficient metal-free electrocatalyst for oxygen reduction in fuel cells. ACS Nano.

[cit25] Wang Y., Shao Y., Matson D. W., Li J., Lin Y. (2010). Nitrogen-doped graphene and its application in electrochemical biosensing. ACS Nano.

[cit26] Shao Y., Zhang S., Engelhard M. H., Li G., Shao G., Wang Y., Liu J., Aksay I. A., Lin Y. (2010). Nitrogen-doped graphene and its electrochemical applications. J. Mater. Chem..

[cit27] Miele G., Bellucci S., Cataldo A., Di Tinno A., Ferrigno L., Maffucci A., Micheli L., Sibilia S. (2021). Electrical impedance spectroscopy for real-time monitoring of the life cycle of graphene nanoplatelets filters for some organic industrial pollutants. IEEE Trans. Instrum. Meas..

[cit28] Bellucci S., Maffucci A., Maksimenko S., Micciulla F., Migliore M. D., Paddubskaya A., Pinchera D., Schettino F. (2018). Electrical permittivity and conductivity of a graphene nanoplatelet contact in the microwave range. Materials.

[cit29] Ferrigno L., Cataldo A., Sibilia S., Maffucci A., Bellucci S. (2020). A monitorable and renewable pollution filter based on graphene nanoplatelets. Nanotechnology.

[cit30] Potenza M., Cataldo A., Bovesecchi G., Corasaniti S., Coppa P., Bellucci S. (2017). Graphene nanoplatelets: Thermal diffusivity and thermal conductivity by the flash method. AIP Adv..

[cit31] Cabrera H., Korte D., Budasheva H., Abbasgholi N. A., Asbaghi B., Bellucci S. (2021). Through-plane and in-plane thermal diffusivity determination of graphene nanoplatelets by photothermal beam deflection spectrometry. Materials.

[cit32] Kukhta A. V., Paddubskaya A. G., Kuzhir P. P., Maksimenko S. A., Vorobyova S. A., Bistarelli S., Cataldo A., Bellucci S. (2016). Copper nanoparticles decorated graphene nanoplatelets and composites with PEDOT:PSS. Synth. Met..

[cit33] Wu Y., Chen M., Chen M., Ran Z., Zhu C., Liao H. (2017). The reinforcing effect of polydopamine functionalized graphene nanoplatelets on the mechanical properties of epoxy resins at cryogenic temperature. Polym. Test..

[cit34] Cha I., Lee E. J., Park H. S., Kim J. H., Kim Y. H., Song C. (2014). Facile electrochemical synthesis of polydopamine-incorporated graphene oxide/PEDOT hybrid thin films for pseudocapacitive behaviors. Synth. Met..

[cit35] Zhang L., Yin M., Wei X., Sun Y., Chen Y., Qi S., Xu D. (2022). Synthesis of rGO@ PDA@ AuNPs for an effective electrochemical chloramphenicol sensor. Diamond Relat. Mater..

[cit36] Sun L., Wang L., Tian C., Tan T., Xie Y., Shi K., Li M., Fu H. (2012). Nitrogen-doped graphene with high nitrogen level *via* a one-step hydrothermal reaction of graphene oxide with urea for superior capacitive energy storage. RSC Adv..

[cit37] Dong Wook C., Hyun-Jung C. (2015). Jong-Beom Baek; Wet-chemical nitrogen-doping of graphene nanoplatelets as electrocatalysts for the oxygen reduction reaction. J. Mater. Chem. A.

[cit38] Lee M. S., Choi H. J., Baek J. B., Chang D. W. (2017). Simple solution-based synthesis of pyridinic-rich nitrogen-doped graphene nanoplatelets for supercapacitors. Appl. Energy.

[cit39] Chang D. W., Lee E. K., Park E. Y., Yu H., Choi H. J., Jeon I. Y., Baek J. B. (2013). Nitrogen-doped graphene nanoplatelets from simple solution edge-functionalization for n-type field-effect transistors. J. Am. Chem. Soc..

[cit40] Madbouly L. A., Sturm H., Doolin A., Hodoroaba V. D., Radnik J. (2025). Chemical analysis of commercial functionalized graphene nanoplatelets along the production process with Raman spectroscopy and X-ray photoelectron spectroscopy. J. Phys. Chem. C.

[cit41] Sheng Z. H., Shao L., Chen J. J., Bao W. J., Wang F. B., Xia X. H. (2011). Catalyst-free synthesis of nitrogen-doped graphene via thermal annealing graphite oxide with melamine and its excellent electrocatalysis. ACS Nano.

[cit42] Chen G.-J., Ng K. Y. S., Lin C.-C. (2022). Effects of nitrogen-doping or alumina films on graphene as anode materials of lithium-ion batteries verified by in situ XRD. J. Nanomater..

[cit43] Ali A., Chiang Y. W., Santos R. M. (2022). X-ray diffraction techniques for mineral characterization: A review for engineers of the fundamentals, applications, and research directions. Minerals.

[cit44] Jorio A. , Saito R., Dresselhaus G. and Dresselhaus M. S., Summary of Raman spectroscopy on sp2 nanocarbons, in Raman Spectroscopy in Graphene Related Systems, ed. A. Jorio, R. Saito, G. Dresselhaus and M. S. Dresselhaus, Wiley-VCH, 2011, 10.1002/9783527632695.ch14

[cit45] Bishop J. L., Pieters C. M., Edwards J. O. (1994). Infrared spectroscopic analyses on the nature of water in montmorillonite. Clays Clay Miner..

[cit46] Page A. J., Chou C.-P., Pham B. Q., Witek H. A., Irle S., Morokuma K. (2013). Quantum chemical investigation of epoxide and ether groups in graphene oxide and their vibrational spectra. Phys. Chem. Chem. Phys..

[cit47] Milosavljevic V., Mitrevska K., Adam V. (2022). Benefits of oxidation and size reduction of graphene/graphene oxide nanoparticles in biosensing application: Classification of graphene/graphene oxide nanoparticles. Sens. Actuators, B.

[cit48] Buzzati-Traverso A. A. , Perspectives in Marine Biology, Univ of California Press, 1958, 10.1016/0003-9861(59)90115-8

[cit49] Ray P., Gidley D., Badding J. V., Lueking A. D. (2019). UV and chemical modifications of polymer of intrinsic microporosity 1 to develop vibrational spectroscopic probes of surface chemistry and porosity. Microporous Mesoporous Mater..

[cit50] Van Hoozen B. L., Petersen P. B. (2018). Vibrational tug-of-war: The p*K*_a_ dependence of the broad vibrational features of strongly hydrogen-bonded carboxylic acids. J. Chem. Phys..

[cit51] Tene T., Guevara M., Benalcázar Palacios F., Morocho Barrionuevo T. P., Vacacela Gomez C., Bellucci S. (2023). Optical properties of graphene oxide. Front. Chem..

[cit52] Ghulam A. N., dos Santos O. A. L., Hazeem L., Pizzorno Backx B., Bououdina M., Bellucci S. (2022). Graphene oxide (GO) materials: Applications and toxicity on living organisms and environment. J. Funct. Biomater..

[cit53] Tene T., Bellucci S., Guevara M., Arias Arias F., Sáez Paguay M. Á., Quispillo Moyota J. M., Arias Polanco M., Scarcello A., Vacacela Gomez C., Straface S., Caputi L. S., Torres F. J. (2022). Adsorption of mercury on oxidized graphenes. Nanomaterials.

[cit54] Dong X., Fu D., Fang W., Shi Y., Chen P., Li L.-J. (2009). Doping single-layer graphene with aromatic molecules. Small.

[cit55] Casimir D. , Alghamdi H., Ahmed I. Y., Garcia-Sanchez R. and Misra P., Raman spectroscopy of graphene, graphite and graphene nanoplatelets, in 2D Materials, IntechOpen, 2019, 10.5772/intechopen.84527

[cit56] Gupta A., Chen G., Joshi P., Tadigadapa S., Eklund P. C. (2006). Raman scattering from high-frequency phonons in supported n-graphene layer films. Nano Lett..

[cit57] Morsin M., Isaak S., Morsin M., Yusof Y. (2017). Characterization of defect induced multilayer graphene. Int. J. Electr. Comput. Eng..

[cit58] Nor N. H. M., Anuar N. A., Talik N. A., Wan W. A. T., Abdullah K. K., Nakajima H., Zaman M. F. (2023). Effects of high-energy electron beam irradiation on the structure, composition and morphological properties of graphene nanoplatelet films. Sains Malays..

[cit59] Iqbal W., Ur Rehman N., Wali N. (2025). Synthesis and structural analysis of graphene nanosheets via microwave atmospheric pressure plasma. Int. J. Mod. Phys. B.

[cit60] Pyykkö P., Atsumi M. (2009). Chem.–Eur. J..

[cit61] Zhao Y. (2011). et al.. Science.

[cit62] Ketabi N., de Boer T., Karakaya M., Zhu J., Podila R., Rao A. M., Kurmaev E. Z., Moewes A. (2016). RSC Adv..

[cit63] Biesinger M. C. (2022). Accessing the robustness of adventitious carbon for charge referencing (correction) purposes in XPS analysis: Insights from a multi-user facility data review. Appl. Surf. Sci..

[cit64] Yamamoto S. (2008). *et al.*, O 1s X-ray photoelectron spectroscopy of oxygen-containing species on carbon surfaces. J. Phys.:Condens. Matter.

[cit65] Moulder J. F. , Stickle W. F., Sobol P. E. and Bomben K. D., Handbook of X-Ray Photoelectron Spectroscopy: A Reference Book of Standard Spectra for Identification and Interpretation of XPS Data, Perkin-Elmer Corporation, Eden Prairie, USA, 1992

[cit66] Lowell S. , Shields J. E., Thomas M. A. and Thommes M., Characterization of Porous Solids and Powders: Surface Area, Pore Size and Density, Springer Science & Business Media, 2012, vol. 16

[cit67] Tian X., Itkis M. E., Bekyarova E., Haddon R. C. (2013). Anisotropic thermal and electrical properties of thin thermal interface layers of graphite nanoplatelet-based composites. Sci. Rep..

[cit68] Peng W., Li H., Liu Y., Song S. (2017). A review on heavy metal ions adsorption from water by graphene oxide and its composites. J. Mol. Liq..

[cit69] Hsine Z., Mlika R., Jaffrezic-Renault N., Korri-Youssoufi H. (2022). Review-Recent progress in graphene based modified electrodes for electrochemical detection of dopamine. Chemosensors.

